# The role of branched-chain aminotransferase 1 in driving glioblastoma cell proliferation and invasion varies with tumor subtype

**DOI:** 10.1093/noajnl/vdad120

**Published:** 2023-09-16

**Authors:** Maria Fala, Susana Ros, Ashley Sawle, Jyotsna U Rao, Anastasia Tsyben, Laura Tronci, Christian Frezza, Richard Mair, Kevin M Brindle

**Affiliations:** Cancer Research UK Cambridge Institute, University of Cambridge, Cambridge, United Kingdom; Cancer Research UK Cambridge Institute, University of Cambridge, Cambridge, United Kingdom; Cancer Research UK Cambridge Institute, University of Cambridge, Cambridge, United Kingdom; Cancer Research UK Cambridge Institute, University of Cambridge, Cambridge, United Kingdom; Cancer Research UK Cambridge Institute, University of Cambridge, Cambridge, United Kingdom; MRC Cancer Unit, University of Cambridge, Hutchison/MRC Research Centre, Cambridge, United Kingdom; MRC Cancer Unit, University of Cambridge, Hutchison/MRC Research Centre, Cambridge, United Kingdom; Cancer Research UK Cambridge Institute, University of Cambridge, Cambridge, United Kingdom; Cancer Research UK Cambridge Institute, University of Cambridge, Cambridge, United Kingdom; Department of Biochemistry, University of Cambridge, Cambridge, United Kingdom

**Keywords:** alpha-ketoglutarate, branched-chain aminotransferase, glioblastoma, hypoxia-inducible factor, proliferation

## Abstract

**Background:**

Branched-chain aminotransferase 1 (BCAT1) has been proposed to drive proliferation and invasion of isocitrate dehydrogenase (*IDH*) wild-type glioblastoma cells. However, the Cancer Genome Atlas (TCGA) dataset shows considerable variation in the expression of this enzyme in glioblastoma. The aim of this study was to determine the role of BCAT1 in driving the proliferation and invasion of glioblastoma cells and xenografts that have widely differing levels of BCAT1 expression and the mechanism responsible.

**Methods:**

The activity of BCAT1 was modulated in *IDH* wild-type patient-derived glioblastoma cell lines, and in orthotopically implanted tumors derived from these cells, to examine the effects of BCAT1 expression on tumor phenotype.

**Results:**

In cells with constitutively high BCAT1 expression and a glycolytic metabolic phenotype, inducible shRNA knockdown of the enzyme resulted in reduced proliferation and invasion by increasing the concentration of α-ketoglutarate, leading to reduced DNA methylation, HIF-1α destabilization, and reduced expression of the transcription factor Forkhead box protein M1 (FOXM1). Conversely, overexpression of the enzyme increased HIF-1α expression and promoted proliferation and invasion. However, in cells with an oxidative phenotype and very low constitutive expression of BCAT1 increased expression of the enzyme had no effect on invasion and reduced cell proliferation. This occurred despite an increase in HIF-1α levels and could be explained by decreased TCA cycle flux.

**Conclusions:**

There is a wide variation in BCAT1 expression in glioblastoma and its role in proliferation and invasion is dependent on tumor subtype.

**Key Points**

- Branched-chain aminotransferase 1 (BCAT1) has been proposed to drive cell proliferation and invasion in *IDH* wild-type glioblastoma.
- BCAT1 is not uniformly upregulated in *IDH* wild-type glioblastoma.
- Increased BCAT1 expression promotes proliferation and invasion in glioblastoma with constitutively high levels of BCAT1 expression, via HIF stabilization, but inhibits proliferation in cells expressing low levels.

**Importance of the Study**

Branched-chain aminotransferase 1 has been proposed to drive cell proliferation and invasion in glioblastoma, although the mechanisms responsible are unclear. However, the wide variation in expression of the enzyme, with some tumors showing very low levels of expression, questions the importance of its role. We show here that increasing expression of the enzyme in cells with preexisting high levels of expression promotes proliferation through stabilization of HIF-1α. However, increasing expression in cells with low levels of expression inhibits proliferation, which has implications for the ongoing development of drugs that target this enzyme.

The branched-chain amino acids (BCAAs) leucine, isoleucine, and valine are essential amino acids that are converted to their respective ketoacids (BCKAs) via transamination of α-ketoglutarate (α-KG) to produce glutamate, catalyzed by Branched Chain Aminotransferase, which exists as cytosolic (BCAT1) and mitochondrial (BCAT2) isoforms (Figure 1A). Upregulation of BCAT1 has been observed in various cancers,^1–13^ where in some cases it has been linked to increased cell proliferation and invasion. However, this is dependent on tissue of origin, for example, BCAT1 has been shown to be required for tumor formation in non-small-cell lung cancer but not in pancreatic ductal adenocarcinoma.^14^ Moreover, different mechanisms have been proposed to be responsible for this association. In chronic myeloid leukaemia (CML) increased BCAT1 expression drives progression by increasing the production of BCAAs^15^ whereas in acute myeloid leukaemia (AML) it drives proliferation by lowering the concentration of α-KG.^5^ Increased expression of BCAT1 has been observed in *IDH* wild-type glioblastoma, but not in gliomas harboring mutations in *IDH1* and *IDH2*.^7^ This was proposed to promote disease progression by increasing the production of Tricarboxylic Acid (TCA) cycle intermediates for biosynthetic processes^7^ and by inhibiting macrophage phagocytic activity through the secretion of BCKAs.^6^

**Figure 1.**
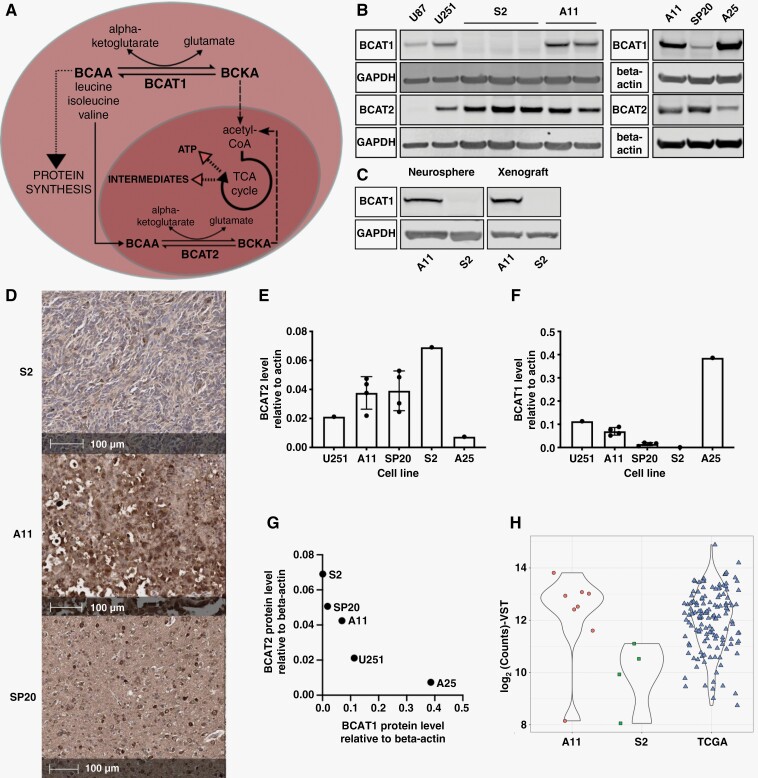
BCAT1 concentrations vary between patient-derived glioblastoma cell lines. (A) The transamination reactions catalyzed by BCAT1 and BCAT2. (B) Representative western blots for BCAT1 and BCAT2 in cell lysates. GAPDH and β-actin were used as loading controls. (C) Representative western blots for BCAT1 in neurospheres and orthotopic xenografts in rats. GAPDH was used as a loading control. (D) Representative immunohistochemical images of orthotopic xenografts in rats derived from S2, A11, and SP20 cells, showing BCAT1 staining. Quantitation of BCAT2 (E) and BCAT1 (F) protein expression in cell lysates. Each point represents a biological replicate and error bars represent Standard Deviations. (G) Mean BCAT1 and BCAT2 protein concentrations relative to β-actin for each of the cell lines as measured by western blot. (H) Comparison of BCAT1 expression levels in A11 and S2 xenografts with TCGA data from IDH wild-type glioblastoma tumors.

However, examination of the Cancer Genome Atlas (TCGA) data shows that there are a wide variation in BCAT1 expression in *IDH* wild-type glioblastoma. Here we have investigated the mechanisms by which increased BCAT1 activity promotes glioblastoma progression by modulating the activity of the enzyme in patient-derived glioblastoma cells with high and very low levels of BCAT1 expression and in mouse and rat orthotopic tumor models derived from them.

## Materials and Methods

### Cell Culture

Cells were mycoplasma tested and authenticated. Human glioma cell lines, U87 (RRID:CVCL_0022; https://scicrunch.org/resolver/CVCL_0022 and https://web.expasy.org/cellosaurus/CVCL_0022) and U-251MG (RRID:CVCL_2219 https://scicrunch.org/resolver/RRID:CVCL_2219 and https://web.expasy.org/cellosaurus/CVCL_0021) (ATCC), were grown in DMEM (Cat# 21969035, Gibco,) supplemented with 10% FBS. Patient-derived glioblastoma cell lines previously isolated from patient samples, as described in,^16^ were cultured in serum-free Neurobasal Medium (Cat# 12349015, Gibco) supplemented with B27 (Cat#17504044, Gibco), N2 (Cat# A1370701, ThermoFisher Scientific), 20 ng/mL EGF (Cat# E9644, Sigma), 20 ng/mL FGF (Cat # PHG0261, Gibco), 2 mM glutamine and Penicillin-Streptomycin (100 U/ml) (Cat# 15070063, Gibco) in flasks precoated with Extracellular Matrix (Cat# E1270, Sigma). For hypoxia ­experiments, cells were incubated in a Tri-Gas incubator (1% O_2_, 5% CO_2_) or in a hypoxia chamber (0.1% or 1% O_2,_ 5% CO_2_).

### Orthotopic Tumor Models

Procedures were performed in compliance with licenses issued under the United Kingdom Animals Scientific Procedures Act, 1986 and approved by an ethical review body. Cells were implanted orthotopically^16^ in 12-week-old (20 g) BALB/c nude mice and 12-week-old (200 g) RNU rats (Charles River,). Randomization was achieved by implanting the different cell lines in mice or rats coming from the same cage. Tumor growth was monitored using MRI (Supplementary Information). For doxycycline treatment, animals were fed a diet containing 200 ppm doxycycline (Cat# TD.180625, Envigo) for 10 days. At least 3 animals were implanted for each condition and samples were only excluded in those cases where the animals had to be culled due to development of symptoms prior to the endpoint. Investigators were not blinded to group allocations.

### Western Blots

Protein extracts were prepared from cells and homogenized tissue using cold Pierce RIPA buffer (Cat#89901, Thermo Fisher Scientific, Waltham,) containing EDTA-free Protease Inhibitor Cocktail (Roche, Basel, Switzerland). Samples were run on 4%–12% Bis-Tris precast polyacrylamide gels (NuPAGE) and transferred onto a nitrocellulose membrane using dry (iBlot 2 Dry Blotting System [Thermofisher]) or wet (NuPAGE) transfer. Primary antibody diluents (Supplementary Information) were prepared in Odyssey Blocking Buffer (Licor) with 0.2% Tween-20 or in 5% Bovine Serum Albumin in Tris-Buffered Saline with 0.1% Tween-20.

### Immunohistochemistry

Rat brains were dissected and fixed in 10% neutral buffered formalin for 24 hours and transferred to 70% ethanol for processing into paraffin blocks. The antibodies used are shown in Supplementary Information and stained sections were imaged using Leica’s Polymer Refine Detection System on the Bond-III platform.

### BCAT Activity Assay

Cells were lysed in cold lysis buffer (50 mM HEPES, 1 mM EDTA, 0.7 % sodium deoxycholate, 1 % Nonidet P-40, 0.5 M lithium chloride, pH 7.6 with EDTA-free Protease Inhibitor Cocktail (Roche)) and assayed as described in.^17^ The reaction (5 mM leucine, 5 mM α-KG, 5 mM ammonium sulphate, 0.05 mM NADH, 0.5 mM GTP, 1 mM DTT, 1.9 U leucine dehydrogenase and 5–20 μL of cell extract in 100 mM potassium phosphate buffer (pH 7.4) in a final volume of 200 μL) was conducted in a UV-transparent 96-well plate (Corning) at 37°C and 340 nm absorbance measured in a Clariostar microplate reader (BMG Labtech). The BCAT1 inhibitor, gabapentin (Cat# PHR1049, Sigma-Aldrich), was used to determine the contributions of BCAT1 and BCAT2 to total activity.

### Reverse Transcriptase Quantitative PCR

RNA was extracted using a Qiagen RNA isolation kit (Rneasy Mini Kit, Cat#74104) and quantified using a Qubit RNA BR Assay Kit (Cat# Q10210 ThermoFisher Scientific). Reverse transcription was performed using M-MuLV Reverse Transcriptase (NEB) and quantitative PCR was performed using Fast SYBR Green master mix (Cat#4385610, Applied Biosystems). QuantStudio software (Applied Biosystems) was used for analysis.

### BCAT1 and BCAT2 Knockdowns

An shRNA sequence targeting BCAT1 (shBCAT1) or a control sequence (shScr) (Supplementary Information) were cloned into the pLKO.1-Tet-OnTet-pLKO-puro lentiviral plasmid^18^ (Addgene plasmid # 21915; http://n2t.net/addgene:21915; RRID:Addgene_21915; https://scicrunch.org/scicrunch/resolver/RRID:Addgene_21915; http://www.addgene.org/21915). Plasmids psPAX2 (7.5 μg) and D2G (2.5 μg) (Addgene plasmid # 12260; https://scicrunch.org/resolver/Addgene_12260/; http://n2t.net/addgene:12260; RRID:Addgene_12260 http://www.addgene.org/12260) and Addgene plasmid # 12259; http://n2t.net/addgene:12259; RRID:Addgene_12259 http://www.addgene.org/12259; https://scicrunch.org/resolver/Addgene_12259/) and 10 μg of the lentiviral plasmid were mixed with 500 μL serum-free DMEM and added to 80 μL Lipofectamine 3000 Transfection Reagent in 500 μL serum-free DMEM and the solution was added to HEK293 cells (ThermoFisher Scientific). Cell medium was filtered through a 0.45 μm filter and 1 mL of this was used to infect glioblastoma cells with 8 μL of the 5 mg/mL polybrene solution. Infected cells were selected with 2 μg/mL puromycin (Gibco). Fifty ng/ml Doxycycline Hyclate (Sigma) was used to induce knockdown.

### BCAT1 Overexpression

The coding regions of human BCAT1 or luciferase were subcloned from a GeneArt plasmid (Thermo Fisher Scientific) into a pBOBI plasmid (gift from Verma laboratory, Salk Institute La Jolla), which contains an EF1 promoter used to drive the expression of mStrawberry and BCAT1 or mStrawberry and luciferase, respectively. Lentiviruses were produced following transfection of HEK293 cells with the plasmids as described above using pMDL packaging plasmid (Addgene #12251; RRID:Addgene_12251 (https://scicrunch.org/resolver/Addgene_12251/mentions?q=&i=rrid:addgene_12251 and http://www.addgene.org/12251), pCMV-VSV-G envelope vector (Addgene #8454; RRID:Addgene_8454 https://scicrunch.org/resolver/Addgene_8454 and http://www.addgene.org/8454) and pRSV-Rev (RRID: Addgene #12253; https://scicrunch.org/resolver/Addgene_12253/ and http://www.addgene.org/12253) and were used for infection of A11 and S2 cells. Infected cells were FACS sorted and expanded to form BCAT1 overexpressing cell lines or luciferase-overexpressing control cell lines.

### Cell Proliferation

Percentage confluence over time was measured in an Incucyte system (Sartorius, Göttingen, Germany). Cell proliferation was also measured using a luciferase-based assay (Cat#G9711, RealTime Glo, Promega).

### Three-Dimensional Spheroid Invasion Assays

Cells were seeded in Ultra Low attachment 96-well plates (Corning) (4000 cells/well) and an invasion assay was executed as described previously.^19^ Images were acquired with an Incucyte microscope (Sartorius, Göttingen, Germany) and analyzed using a Python script (Supplementary Information).

### Cell Cycle Analysis

Cells (10^6^) were fixed in 4.5 mL ice-cold 70% ethanol, centrifuged (5 minutes, 300 *g*), and washed with PBS. The pellets were resuspended in 600 μL of Propidium Iodide Solution (0.1% Triton X, 2 mg RNAse, 20 mg/mL Propidium Iodide in PBS) and incubated at 37°C for 15 minutes with gentle shaking and then filtered and analyzed on an LSRII (BD) flow cytometer. FlowJo software was used for data analysis.

### Measurements of Oxygen Consumption

Cell oxygen consumption rates were measured using a Seahorse XF96 Extracellular Flux Analyzer (Agilent).

### Limiting Dilution Assay

A11 cells expressing doxycycline-inducible shScr and shBCAT1 were treated with doxycycline for 6 days before washing and staining with 5 μg/mL DAPI. Live cells were sorted on an Influx cell sorter (BD Biosciences, New Jersey). 1, 5, 10, or 20 cells were seeded per well in a 96-well plate containing complete Neurobasal Medium and incubated for 3–4 weeks. The number of tumor sphere-containing wells per group was counted and data were analyzed using Extreme Limiting Dilution Analysis software.^20^

### RNA Sequencing

Cells were treated with doxycycline or vehicle for 7 days before RNA extraction using a RNeasy Mini Kit (Cat#74104, Qiagen). For tumor samples, the whole brain was frozen in liquid nitrogen and the tumor was extracted using a cryostat and surgical scalpel cooled with dry ice. The tissue was homogenized and RNA was extracted using a RNeasy Mini Kit (Qiagen). RNA was quantified using an Agilent 4200 TapeStation. For library preparation, the Illumina Truseq stranded mRNA kit was used, and single-read sequencing was performed on a HiSeq 4000 machine (Illumina). Data analysis were performed as described in Supplementary Information.

### Intracellular Metabolite Analysis

Cells were washed with ice-cold PBS and incubated on dry ice with 500 μL extraction solution (50% Methanol, 30% Acetonitrile, 20% Ultrapure Water, d8-Valine at a final concentration of 5 μM) per million cells added to each well for 15 minutes. Cells were scraped from the wells, agitated (15 minutes, 4°C), incubated for 1 hour at −20°C, vortexed, and centrifuged (21 000 *g*, 10 minutes, 4°C). Liquid Chromatography Mass Spectrometry (LCMS) was used to quantify the relative concentrations of each metabolite of interest and the absolute αKG concentration, as described in Supplementary Information.

### DNA Methylation Analysis

DNA was extracted using a Purelink Genomic DNA Mini Kit (Invitrogen). DNA (1 μg) was incubated with 5 units DNA Degradase Plus (Cat# E2020, Zymo Research) (4 hours, 37°C) and samples were analyzed by LCMS as described in Supplementary Information.

### [2-^13^C,^15^N]Leucine Infusion

A11 tumor-bearing mice were infused via a tail vein cannula with a bolus of 300 mg [2-^13^C,^15^N]leucine/g body weight and then a continuous infusion of 0.0069 mg/g body weight min^−1^ for 150 minutes.^21^ At the end of the infusion, mice were sacrificed by cervical dislocation followed by blood collection in EDTA-coated tubes and dissection and flash freezing of the tumor in liquid nitrogen. Blood samples were centrifuged (2000 *g*, 20 minutes) to collect the plasma. Labeled leucine enrichment was measured in plasma and tumor extracts by LCMS, as described in Supplementary Information.

## Results

### BCAT1 Concentrations Vary Between Patient-Derived Glioblastoma Cell Lines

*IDH* wild-type patient-derived (A11, S2, SP20, and A25) and established human (U87 and U251) and rat (C6) glioblastoma cell lines showed variable concentrations of BCAT1 and BCAT2 (Figure 1B–G; Supplementary Figure S1 A–D). SP20 had very low concentrations of BCAT1 with S2 cells showing no detectable protein (Figure 1B) on western blot. When grown as neurospheres S2 cells still showed no detectable BCAT1 protein (Figure 1C). The same protein expression patterns were observed in rat orthotopic xenografts derived from A11, SP20, and S2 cells. Immunohistochemistry showed the highest BCAT1 staining in A11 xenografts with lower staining in SP20 and minimal staining in S2 xenografts (Figure 1D). These expression patterns were confirmed by western blot for the A11 and S2 xenografts (Figure 1C). There was an inverse relationship between BCAT1 and BCAT2 protein concentrations (Figure 1E–G), which was also reflected in the relative activities of the enzymes (Supplementary Figure S1A–C). A11 cells had higher BCAT1 than BCAT2 activity whereas in SP20 and S2 cells the predominant BCAT activity was due to BCAT2 (Supplementary Figure S1A–C). BCAT2 transcript levels were much higher than for BCAT1 with BCAT1 mRNA being undetectable in S2 cells (Supplementary Figure S1D). Examination of the TCGA dataset for *IDH* wild-type glioblastoma also showed a wide variation in the expression levels of BCAT1, with A11 representative of tumors with high expression and S2 tumors with low expression (Figure 1H).

A11, S2, and SP20 patient-derived cells have been described previously^16^ (referred to previously as GB4, GB1, and GB2, respectively) and were shown to recapitulate the biology of the patient tumors from which they were derived. Following orthotopic implantation, they all showed high expression of the glial cell marker GFAP. Exome sequencing showed *PTEN* frameshift mutations in S2 and SP20 tumors, resulting in protein loss and a *PIK3R1* (V73fs) frameshift mutation in A11, that could lead to activation of PI3K. All the models showed activation of the PI3K/Akt pathway, as indicated by phosphorylation of Akt.^16^ S2 also harbors *TP53* and *RB1* mutations that are not found in A11 (Supplementary Figure S1E). Epithelial-to-mesenchymal transition has been shown to drive BCAT1 expression^22^ and RNAseq profiles showed that A11 cells have more mesenchymal features whereas S2 cells appear more neural progenitor cell-like (Supplementary Figure S1F).^23^ A11 cells are more glycolytic in vitro than S2 cells (Supplementary Figure S1G–H) and previous ^13^C MRI studies of hyperpolarized [1-^13^C]pyruvate metabolism showed higher lactate labeling in A11 tumors than in S2 tumors.^16^ A11 tumors also showed higher levels of expression of the glycolytic enzymes, lactate dehydrogenase A and hexokinase 2 and of the monocarboxylate transporters MCT1 and MCT4.^16^

### Expression of BCAT1 Confers Sensitivity to Inhibition of Proliferation With a BCAT1 Inhibitor

**Figure 2.**
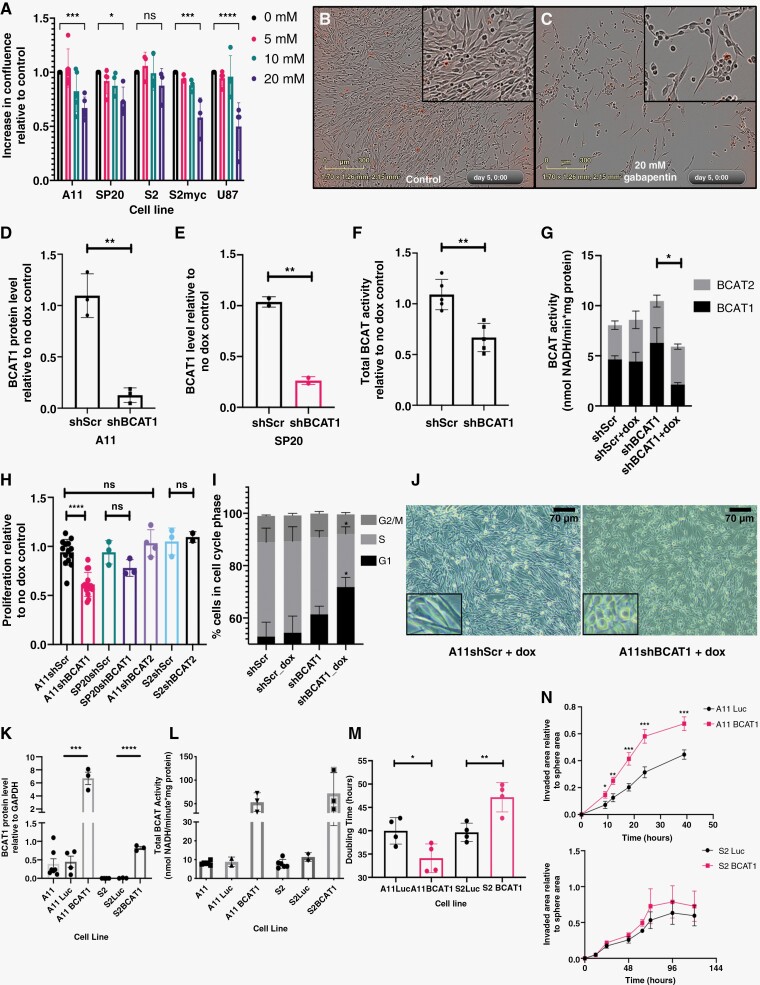
BCAT1 expression promotes proliferation and invasion in A11 cells but not in S2 cells. (A) Degree of cell confluence, relative to control cells, at increasing concentrations of gabapentin. Each point represents a biological replicate, except in the case of U87 where the points represent replicates from a single cell sample. The error bars represent Standard Deviations. Two-tailed *t*-tests were used to compare treated and control groups. Representative images of control A11 cells (B) and A11 cells incubated with 20 mM gabapentin (C). The levels of BCAT1 protein determined by western blot in A11 (D) and SP20 cells (E) expressing a control shRNA (shScr) and shRNA targeting BCAT1 (shBCAT1) relative to their respective no doxycycline treatment controls. Each point represents a biological replicate and error bars represent Standard Deviations. Two-tailed *t*-tests were used to compare the 2 groups. (F) Total BCAT activity in A11 cells expressing shScr and shBCAT1, relative to their respective no doxycycline treatment controls. Each point represents a biological replicate and error bars represent Standard Deviations. Two-tailed *t*-tests were used to compare the 2 groups. (G) Estimated BCAT1 and BCAT2 contributions to the total BCAT activity measured in lysates of A11shScr and A11shBCAT1 cells treated with doxycycline and their respective no-doxycycline treatment controls. Error bars represent the standard error of the mean (*n* = 2). Two-tailed *t*-tests were used to compare the BCAT1 activities in the different cell lines. (H) Proliferation rates for A11shScr, A11shBCAT1, SP20shScr, SP20shBCAT1, A11shBCAT2, S2shScr, and S2shBCAT2 cells, relative to their respective no doxycycline treatment controls measured using the RealTime Glo cell viability luminescence assay. Each point represents the mean relative proliferation rate from an independent experiment and the error bars represent Standard Deviations. Two-tailed *t*-tests were used to compare the relative proliferation rates in the different groups. (I) Summary from 4 independent experiments, showing the relative proportions of A11 cells in each cell cycle phase. Error bars represent standard error of the mean. (J) Representative images of A11shScr and A11shBCAT1 cells following treatment with doxycycline. (K) BCAT1 protein concentration, determined by western blot, relative to GAPDH in A11 and S2 cells, A11 and S2 luciferase-overexpressing control cells and A11 and S2 BCAT1-overexpressing cells. Each point represents a biological replicate and error bars represent standard error of the mean. Two-tailed *t*-tests were used to compare protein expression between the groups. (L) Total BCAT activity in lysates of A11 and S2 cells and in cells overexpressing Luciferase or BCAT1. Each point represents the mean activity from an independent replicate. (M) The doubling times as calculated from fitting growth curves for A11 and S2 luciferase and BCAT1 overexpressing cells to an exponential function. Each point represents the calculated doubling time from an independent experiment. Two-tailed *t*-tests were used to compare the luciferase- and BCAT1-overexpressing cells. (N) Results from 3-dimensional spheroid invasion assays showing the relative invaded area over time for A11 and S2 luciferase- and BCAT1-overexpressing cells in 3 independent experiments. Error bars represent standard error of the mean. Multiple *t*-tests corrected for multiple comparisons using the Holm-Sidak method were used to compare the 2 groups. ns: *P* > .05, *P < .05, **P < .01, ***P < .001, ****P < .0001.

**Figure 3.**
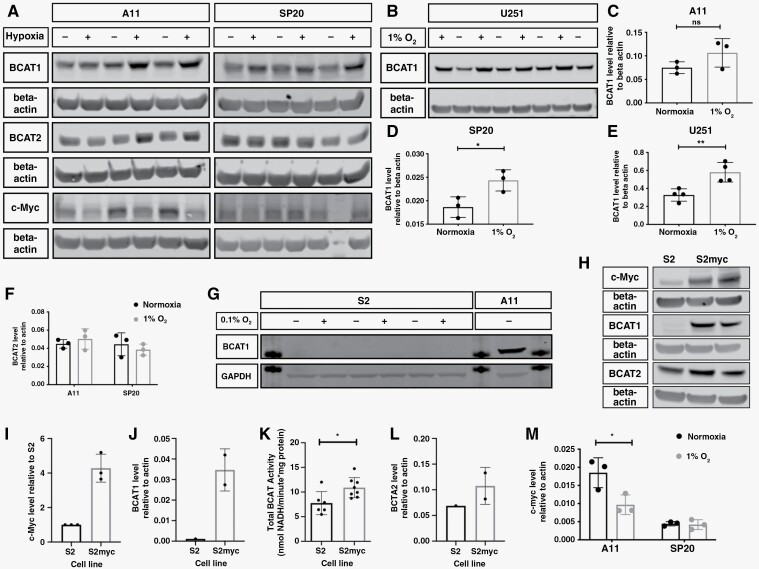
BCAT1 expression is regulated by hypoxia in A11 cells but not in S2 cells where it is regulated by c-Myc. (A) Western blots for BCAT1, BCAT2, and c-Myc in lysates of A11 and SP20 cells grown under normoxia (−) or 1% O_2_ (+) for 6 days. β-actin was used as a loading control. The gap before the last lane in the c-Myc and β-actin blot for SP20 represents the lane where the molecular weight marker was loaded. (B) Western blot for BCAT1 in lysates of U251 cells grown under normoxia (−) or 1% O_2_ (+). β-actin was used as a loading control. Quantitation of BCAT1 protein content, relative to β-actin, in A11, SP20, and U251 cells (C-E) and BCAT2 (F), under normoxic and hypoxic conditions. Each point represents a biological replicate and error bars represent Standard Deviations. Two-tailed *t*-tests were used to compare the expression under normoxic and hypoxic conditions. ns:*P* > .05, *P < .05, **P < .01. (G) Western blot for BCAT1 in lysates of S2 cells grown under normoxia (−) or 0.1% O_2_ (+). GAPDH was used as a loading control. A11 lysates were included as a positive control for BCAT1 expression. The additional bands visible are from the 37 kDa molecular weight marker. (H) Representative western blots for c-Myc, BCAT1, and BCAT2 in lysates of S2 and S2 c-Myc-overexpressing cells. β-actin was used as a loading control. Quantitation of c-Myc (I) and BCAT1 (J) concentrations, total BCAT activity (K) and BCAT2 concentrations (L) in S2 and S2 c-Myc-overexpressing cells. Each point represents a biological replicate and error bars represent standard deviations. (M) c-Myc concentration in A11 and SP20 cells, relative to β-actin, under normoxic and hypoxic conditions. Each point represents a biological replicate and error bars represent Standard Deviations. Two-tailed *t*-tests were performed to compare the cell lines under normoxia and hypoxia (1% O_2_), *P < .05.

Treatment of A11, SP20, S2, and U87 cells with increasing concentrations of gabapentin, a selective BCAT1 inhibitor,^24^ reduced proliferation of A11, SP20, and U87 cells, and changed A11 and SP20 cell morphology, but had no effect on S2 cell proliferation or morphology (Figure 2A–C, Supplementary Figure S2A). The effects of gabapentin were phenocopied by shRNA-mediated knockdown of BCAT1 expression. Knockdown of BCAT1 using a doxycycline-inducible shRNA (shBCAT1) (Figure 2D–G; Supplementary Figure S3 A,D) decreased proliferation of A11 cells but had a smaller effect on the proliferation of SP20 cells (Figure 2H). Knockdown of BCAT2 in A11 or S2 cells (Supplementary Figure S3B) had no effect on cell proliferation (Figure 2H). Knockdown of BCAT1 in A11 cells resulted in partial cell cycle arrest in G1 phase (Figure 2I; Supplementary Figure S3E) and a more rounded morphology (Figure 2Jj), similar to that observed with gabapentin (Figure 2C). There was no effect on cell viability in A11 or SP20 cells upon BCAT1 knockdown (Supplementary Figure S3C). Overexpression of BCAT1 (Figure 2K–Ll; Supplementary Figure S3F) increased cell proliferation and invasion in A11 cells but inhibited proliferation in S2 cells and had no effect on invasion (Figure 2M–N; Supplementary Figure S3G–I). Overexpression of c-Myc in S2 cells (S2myc), which increased BCAT1 expression (Figure 3H–I) and increased their proliferation (Supplementary Figure S2B), conferred sensitivity to gabapentin inhibition of proliferation (Figure 2A), which now also changed S2 cell morphology (Supplementary Figure S2A).

### BCAT1 Expression is Regulated by Hypoxia in A11 Cells But Not in S2 Cells Where it is Regulated by c-Myc

BCAT1 expression is driven by hypoxia^25^ and c-Myc.^26,27^ Incubation of cells in 1% or 0.1% O_2_ increased BCAT1 protein concentration in A11, SP20 and U251 cells (Figure 3A–E) but not in S2 cells (Figure 3G) and had no effect on BCAT2 expression in A11 and SP20 cells (Figure 3F). Overexpression of c-Myc in S2 cells, which had low levels of c-Myc (Figure 3H–I), markedly increased the concentration of BCAT1 (Figure 3J,K) but had no effect on the concentration of BCAT2 (Figure 3L). Increased expression of BCAT1 in hypoxic A11 cells occurred despite a decrease in c-Myc concentration (Figure 3M). In summary, A11, SP20, and U251 cells show upregulation of BCAT1 expression in hypoxia whereas S2 cells display regulation of BCAT1 expression by c-Myc but not by hypoxia. Whole genome sequencing of A11, SP20, and S2 cells performed previously^16^ showed no differences in the coding, promoter, or enhancer regions of the BCAT1 gene with the exception of an intronic variant/mutation in the promoter region in 2 A11 samples.

### BCAT1 Expression Regulates the Concentration of HIF-1α and the Expression of HIF-1α Target Genes

**Figure 4.**
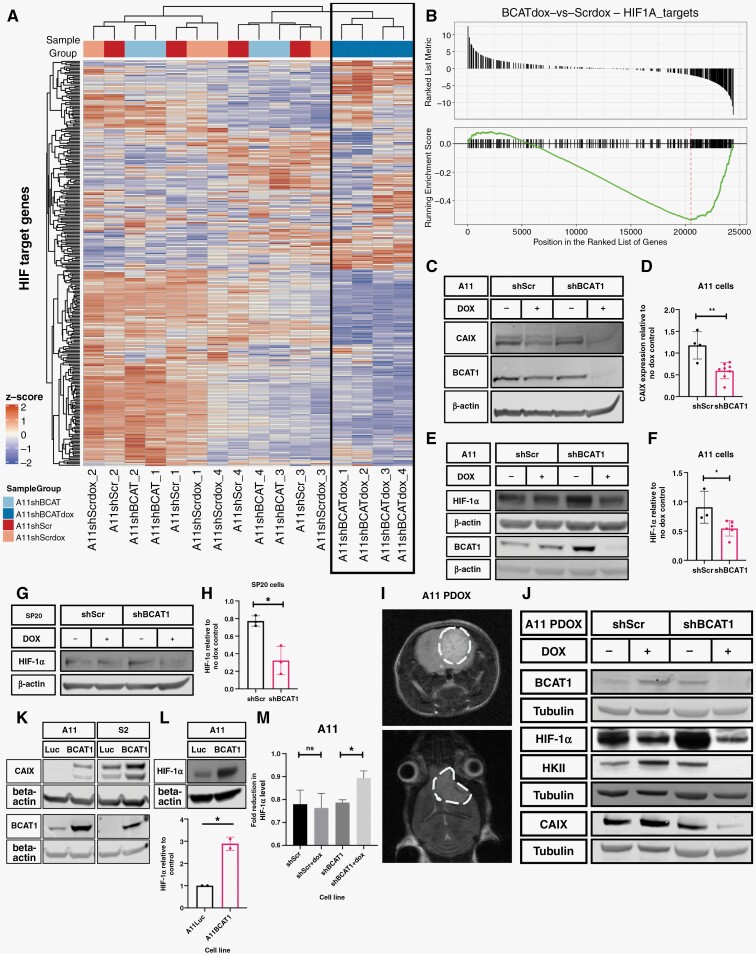
BCAT1 expression regulates the concentration of HIF-1α and the expression of HIF target genes. (A) Heatmap showing the relative expression of HIF target genes in each of 4 biological replicates of A11 cells expressing doxycycline-inducible shScr and shBCAT1 and their respective no doxycycline treatment controls. Results from clustering analysis performed based on the expression of these target genes are shown at the top of the heatmap, with all A11 BCAT1 knockdown samples clustering together and separately from all the control samples. (B) Gene set enrichment analysis of HIF1α targets in A11 BCAT1 knockdown cells compared to control cells. (C) Representative western blot for carbonic anhydrase IX (CAIX) and BCAT1 in cell lysates of A11 cells expressing doxycycline-inducible shScr and shBCAT1 and their non-induced controls. β-actin was used as a loading control. (D) Quantitation of band intensities for CAIX using gel densitometry, where the CAIX/β-actin ratio is expressed as a ratio to the CAIX/β-actin ratio in non-induced controls. Each point represents a biological replicate, and the error bars represent Standard Deviations. Two-tailed *t*-tests were performed to compare the 2 groups, **P < .01. Representative western blots for HIF-1α and BCAT1 in lysates of A11 (E) and SP20 (G) cells expressing doxycycline-inducible shScr or shBCAT1, with β-actin used as a loading control. Quantitation of the band intensities for HIF-1α using gel densitometry, where the HIF-1α/β-actin ratio is expressed as a ratio to the HIF-1α/β-actin ratio in non-induced controls in A11 cells (F) and SP20 cells (H). Each point represents a biological replicate, and the error bars represent Standard Deviations. Two-tailed *t*-tests were performed to compare the 2 groups, *P < .05, **P < .01. For the western blots for HIF-1α, cells were incubated in 1% O_2_ for 8 hours prior to cell lysis. The BCAT1 blot for SP20 lysates is in Supplementary Figure S2A. To assess the effects of BCAT1 knockdown in vivo, mice were implanted orthotopically with A11 cells expressing doxycycline-inducible shScr or shBCAT1. The mice were fed with a doxycycline-containing diet or vehicle diet for 10 days and the tumors were resected and cells lysed for western blot analysis. (I) Representative T_2_-weighted axial and coronal magnetic resonance images of an orthotopic tumor in a mouse brain. (J) Representative western blots for BCAT1, HIF-1α, Hexokinase II (HKII), and CAIX in lysates of orthotopic xenografts from mouse brains. Tubulin was used as a loading control. (K) Representative western blots for CAIX and BCAT1 in A11 and S2 luciferase-overexpressing and BCAT1-overexpressing cells. β-actin was used as a loading control. (L) Representative western blot and quantitation of HIF-1α protein concentration relative to β-actin in A11 luciferase and A11 BCAT1-overexpressing cells from 2 biological replicates. Two-tailed *t*-tests were used to compare relative expression. *P < .05. (M) The stability of HIF-1α in A11 cells expressing doxycycline-inducible shScr and shBCAT1 was assessed by measuring the extent of degradation of the protein when transferring the cells from 1% O_2_ to normoxic conditions for 10 minutes. The cells were induced with doxycycline for 4 days and incubated in 1% O_2_ for 8 hours prior to the experiment. The fold-reduction in HIF-1α relative to β-actin is plotted for each cell line, with error bars representing Standard Error on the Mean from 3 biological replicates. Two-tailed *t*-tests were used to compare the cell lines. ns: *P* > .05, *P < .05.

BCAT1 knockdown in A11 cells changed their transcriptional profile (Supplementary Figure S4), with the expression of cyclins and cell cycle checkpoint proteins being downregulated, consistent with partial cell cycle arrest (Figure 2I). HIF transcriptional targets were also significantly downregulated (Figure 4A–B, Supplementary Figure S4). HIF-1α protein, and the product of a target gene, carbonic anhydrase IX (CAIX),^28^ were significantly downregulated following BCAT1 knockdown in A11 and SP20 cells (Figure 4C–H). HIF-1α, and the products of its target genes, CAIX and hexokinase II (HKII),^29^ were also significantly downregulated following doxycycline-induced BCAT1 knockdown in orthotopically implanted A11 xenografts (Figure 4I–J). Conversely, BCAT1 overexpression in A11 and S2 cells increased the concentrations of HIF-1α and CAIX in A11 cells and CAIX in S2 cells (Figure 4K–L). Following BCAT1 knockdown, A11 cells were incubated in 1% O_2_ for 8 hours and then extracted either immediately or following 10 minutes of incubation in normoxic conditions. BCAT1 knockdown increased the degradation of HIF-1α, indicating that it reduces HIF-1α stability (Figure 4M). Immunohistochemistry (Supplementary Figure S5) showed that A11 tumor BCAT1 expression was co-localized with the expression of the monocarboxylate transporters MCT1 and MCT4, which are also HIF transcriptional targets,^30,31^ and with CAIX.

### Effect of BCAT1 Knockdown on Amino Acid Metabolism, TCA Cycle Flux, and mTORC1 Activity

**Figure 5.**
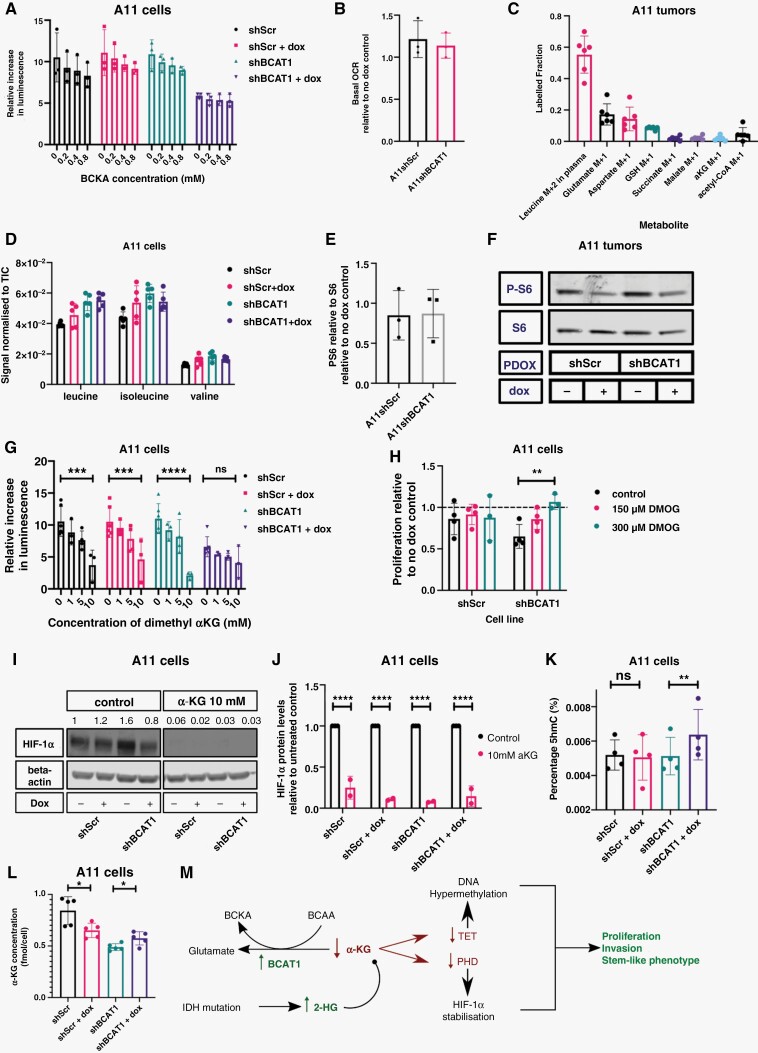
Increased α-ketoglutarate concentrations can explain the effects of BCAT1 knockdown on cell proliferation. (A) Relative rates of cell proliferation at increasing branched-chain ketoacid concentrations. α-ketoisocaproic acid, α-ketoisovaleric acid, and 3-methyl-2-oxopentanoic acid were added at the concentrations indicated. Each point represents the mean increase in luminescence signal in a luciferase-based cell viability assay relative to the initial timepoint, in independent experiments. The error bars represent Standard Deviations. (B) Basal Oxygen Consumption Rate (OCR) in A11 cells expressing doxycycline-inducible shScr and shBCAT1 relative to their respective no doxycycline treatment controls. Each point represents the mean from an independent experiment. Error bars represent Standard Deviations. (C) The fractional isotopic enrichment of the specified metabolites in orthotopic A11 tumors following infusion of [2-^13^C,^15^N]leucine into tumor-bearing mice. Each point represents a biological replicate and error bars represent Standard Deviations. (D) The relative leucine, isoleucine, and valine concentrations in extracts of A11 cells expressing doxycycline-inducible shScr and shBCAT1 and their respective no doxycycline treatment controls. Error bars represent Standard Deviations. (E) The relative levels of P-S6 to S6 ratios in shScr- and shBCAT1-expressing A11 cells, following 48 hours of doxycycline treatment. (F) Western Blot of P-S6 and S6 in orthotopically implanted A11 tumors in mice expressing shScr and shBCAT1. The BCAT1 blot for these lysates is shown in Figure 4J. PDOX: Patient-Derived Orthotopic Xenograft. (G) Proliferation rates, measured using a luciferase-based cell viability assay, of A11 cells expressing doxycycline-inducible shScr and shBCAT1 relative to their respective no doxycycline treatment controls at increasing concentrations of dimethyl α-KG. Each point represents the mean from an independent experiment and the error bars represent Standard Deviations. Two-tailed *t*-tests were used to compare treated cells to controls, ns: *P* > .05, ***P < .001, ****P < .0001. (H) Proliferation rates, measured using a luciferase-based cell viability assay, of A11 cells expressing doxycycline-inducible shScr and shBCAT1 relative to their respective no doxycycline treatment controls at increasing concentrations of dimethyl oxalylglycine (DMOG). Each point represents the mean relative proliferation rate from an independent experiment and the error bars represent Standard Deviations. Two-tailed *t*-tests were used to compare control cells to treated cells, **P < .01. (I) Representative western blot for HIF-1α in A11 cells expressing doxycycline-inducible shScr or shBCAT1. Cells were treated with doxycycline for 96 hours. 10 mM α-KG was added to the cells 24 hours prior to cell lysis, which were incubated at 1% O_2_ for 8 hours prior to cell lysis. β-actin was used as a loading control. The numbers above the HIF-1α bands correspond to their densities relative to the density of the band in the first lane. (J) Quantitation of HIF-1α expression in A11 cells expressing doxycycline-inducible shScr and shBCAT1 and supplemented with 10 mM α-KG, relative to non-doxycycline-treated controls. Each point represents an independent experiment and the error bars represent Standard Deviations. (K) The proportion of 5-hydroxymethylcytosine, as a percentage of total DNA cytosine, in A11 cells expressing doxycycline-inducible shScr and shBCAT1 following incubation with doxycycline for 6 days (no treatment controls are also shown). Each point represents a biological replicate and the error bars represent Standard Deviations. Two-tailed *t*-tests were used to compare the doxycycline-treated cells to the non-doxycycline-treated controls, ns: *P* > .05, **P < .01. (L) LC-MS quantitation of intracellular α-KG in A11 cells expressing doxycycline-inducible shScr and shBCAT1 following 96 hours of doxycycline induction. (M) Proposed mechanism of BCAT1 mediated regulation of α-KG concentration and its effects.

The effect of increased BCAT1 expression on glioblastoma cell proliferation was suggested to result from increased production of BCKAs, from the corresponding BCAAs, and their oxidation in the TCA cycle.^7^ However, inhibition of A11 cell proliferation by BCAT1 knockdown was not relieved by addition of BCKAs (Figure 5A). Moreover, in BCAT1 knockdown cells the basal oxygen consumption rate (Figure 5B) and the rate following addition of the mitochondrial uncoupler FCCP (Supplementary Figure S6A) were not significantly different from control cells, suggesting that BCAT1 knockdown had not reduced flux through the TCA cycle. Infusion of mice bearing orthotopically implanted A11 tumors with [2-^13^C,^15^N]leucine showed minimal incorporation of ^13^C into the TCA cycle intermediates succinate, malate, α-KG, and acetyl-CoA, suggesting that BCAAs are not an important carbon source for the TCA cycle in these tumors (Figure 5C).

Knockdown of BCAT1 in A11 cells resulted in higher concentrations of TCA cycle intermediates, suggesting that BCAT1 knockdown not only does not limit flux into the TCA cycle but actually increases it (Supplementary Figure S6B–E), consistent with downregulation of HIF-1α expression. BCAT1 knockdown also resulted in higher concentrations of glutamate and glutathione (Supplementary Figure S6F–G), contrary to what was observed in an immortalized human astrocyte cell line,^32^ and again consistent with increased flux in the TCA cycle.^33^

BCAT1 expression in CML increases with disease progression and drives the production of BCAAs by aminating the corresponding BCKAs. Knockdown or inhibition of BCAT1 in these cells reduced phosphorylation of S6 kinase, suggesting that the increase in BCAAs, particularly leucine, drives disease progression through activation of mTORC1.^15^ However, there was no evidence that this mechanism drives proliferation of A11 cells. Knockdown of BCAT1 in A11 cells (Supplementary Figure S6H) produced no change in BCAA concentrations (Figure 5D) or in the ratio of phosphorylated to non-phosphorylated S6 (Figure 5E). BCAT1 knockdown also had no effect on the ratio of phosphorylated to non-phosphorylated S6 in orthotopic A11 tumors in mice (Figure 5F). S2 cells overexpressing BCAT1 showed no differences in S6 phosphorylation when compared to S2 control cells (Supplementary Figure S6I).

### Increased α-Ketoglutarate Concentration can Explain the Effects of BCAT1 Knockdown on A11 Cell Proliferation

In AML stem cells BCAT1 upregulation lowers α-KG concentration, resulting in reduced activity of α-KG-dependent dioxygenases, including Ten Eleven Translocation (TET) enzymes and Egl-9 Hypoxia Inducible Factor 1 (Egln1), leading to a hypermethylated DNA state, similar to that in *IDH* mutant cells, and stabilization of HIF-1α, respectively.^5^ Treatment of A11 cells with dimethyl α-KG, a cell-permeable analog of α-KG,^34–36^ mimicked the effects of BCAT1 knockdown on cell proliferation (Figure 5G). Conversely, supplementation with dimethyl oxalyl glycine (DMOG), a competitive inhibitor of α-KG-dependent dioxygenases,^37,38^ restored the proliferation rate of BCAT1 knockdown cells to control levels (Figure 5H). Supplementation of the growth medium with α-KG reduced HIF-1α levels, confirming the role of α-KG in destabilizing HIF-1α in these cells (Figure 5I,J). BCAT1 knockdown increased the levels of 5-hydroxymethylcytosine in DNA, indicating activation of the TET enzymes and supporting the proposal that BCAT1 knockdown leads to the accumulation of α-KG and increased activity of α-KG-dependent dioxygenases (Figure 5K). There was a small increase in α-KG concentration following doxycycline induction of shBCAT1 expression. However, this may be an underestimate of the effect of BCAT1 knockdown since the addition of doxycycline to cells expressing a control shRNA (shScr) depressed the α-KG concentration (Figure 5L). Moreover, the relative change in the cytosolic concentration could be much larger depending on the contribution of α-KG in the mitochondria.

In summary, the effects of changes in BCAT1 activity on A11 cell proliferation are mediated via changes in α-KG concentration. Decreases in BCAT1 activity raise α-KG concentration, leading to increased prolyl hydroxylase activity and HIF-1α destabilization, resulting in inhibition of cell proliferation and invasion. The increase in α-KG concentration also leads to an increase in TET activity resulting in DNA demethylation, as reflected in the increased levels of 5hmC (Figure 5M).

### BCAT1 Knockdown Downregulates Expression of FOXM1 and Inhibits Neurosphere Formation

**Figure 6.**
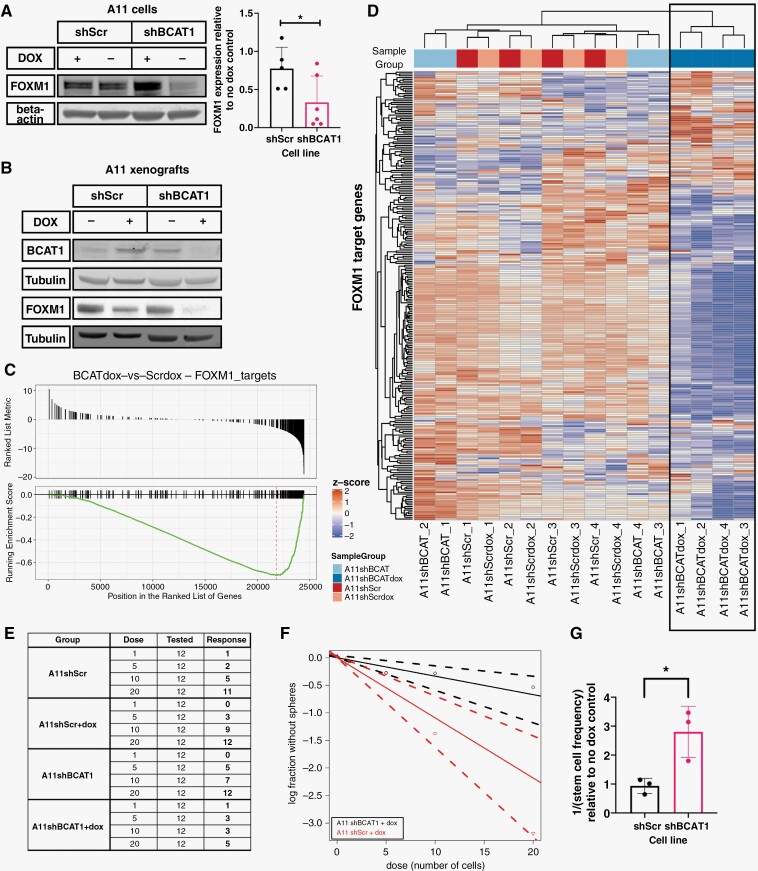
BCAT1 knockdown downregulates expression of the transcription factor FOXM1 and inhibits neurosphere formation. (A) Representative western blot for FOXM1 and quantitation of FOXM1 expression in A11 cells expressing doxycycline-inducible shScr and shBCAT1. β-actin was used as a loading control. The BCAT1 blot for these lysates is shown in Figure 4E. Each point on the quantitation plot represents a biological replicate and the error bars represent Standard Deviations. (B) Representative western blot for BCAT1 and FOXM1 in extracts of orthotopic xenografts grown in mouse brain. Tubulin was used as a loading control. (C) Gene set enrichment analysis of FOXM1 targets in A11 BCAT1 knockdown cells compared to control cells. (D) Heatmap showing the relative expression of FOXM1 target genes in each of 4 biological replicates of A11 cells expressing doxycycline-inducible shScr and shBCAT1 and their respective no doxycycline treatment controls. Results from a clustering analysis based on the expression of these target genes are shown at the top of the heatmap, with all A11 BCAT1 knockdown samples clustering together and separately from the control samples. (E–G) Assessment of stem cell frequency using a tumor sphere formation assay. (E) A representative example of the data collected from a tumor sphere formation assay, where dose is the number of viable cells seeded in each well, tested is the number of replicate wells for the condition and response is the number of wells with a positive result, ie, the presence of a sphere at the endpoint. The data for A11 cells expressing doxycycline-inducible shScr and shBCAT1 are plotted in (F) to show the response of the cells at different cell doses. (G) Estimated stem cell frequency for the A11 cells expressing doxycycline-inducible shScr and shBCAT1 relative to their respective no-doxycycline treatment controls, from 3 independent experiments with the error bars representing Standard Deviations. For (A) and (G), 2-tailed *t*-tests were performed to compare the 2 groups, *P < .05.

Expression of Forkhead box protein M1 (FOXM1), a downstream target of HIF-1α^39^ that has been shown to play multiple roles in glioblastoma,^40–44^ was downregulated following BCAT1 knockdown (Figure 6A–B) and this was accompanied by downregulated expression of its target genes (Figure 6C–D). In addition to promoting cell proliferation, FOXM1 has been implicated in maintaining a stem-like phenotype and the tumorigenicity of glioblastoma cells.^40,43,45,46^ BCAT1 knockdown in A11 cells reduced their capacity to form tumor spheroids and resulted in a significantly reduced frequency of cells with self-renewal capacity (Figure 6E–G).

## Discussion

Increased BCAT1 expression has been described in breast,^47^ ovarian,^9^ and liver cancer,^8^ and in AML^5^ and CML.^15^ However, its role in disease progression varies between different cancer types.^14^ BCAT1 is upregulated in a large proportion of *IDH* wild-type glioblastoma^3,7,48^ and has been identified as one of 4 independent prognostic markers of the disease,^49,50^ although the TCGA dataset shows that there is a wide variation in the levels of expression.

The effect of increased BCAT1 expression on glioblastoma growth was suggested previously to be due to increased flow of BCKAs into the TCA cycle and the production of biosynthetic intermediates.^7^ However, we observed here that increased concentrations of BCKAs were unable to rescue the growth defect caused by knockdown of BCAT1 expression in A11 cells. Moreover, knockdown of BCAT2, which is thought to form a complex with the Branched Chain Ketoacid Dehydrogenase Complex^51^ and is directly involved in BCKA oxidation, had no effect on cell proliferation. Infusion of labeled leucine into tumor-bearing mice resulted in minimal labeling of tumor TCA cycle intermediates, suggesting that these tumors do not rely on BCAAs as a carbon source for the TCA cycle.

An alternative mechanism whereby increased BCAT1 expression can drive disease progression has been described in CML, where net flux in the enzyme-catalyzed reaction is in the opposite direction, resulting in the production of BCAAs from BCKAs. The increased growth with increased BCAT1 expression in CML could be explained by elevated BCAA concentrations, particularly leucine, resulting in activation of the mTORC1 pathway.^15^ Similar observations have been made in human hepatocellular carcinomas and animal models of liver cancer.^52^ However, in A11 cells knockdown of BCAT1 and in S2 cells increased BCAT1 expression had no effect on mTORC1 activity, as evidenced by the absence of a change in phospho-S6 concentration.

The mechanism by which increased BCAT1 expression drives the progression of the glioblastoma subtype represented by A11 has been shown here to be similar to that observed in AML stem cells.^5^ Knockdown of BCAT1 resulted in the accumulation of α-KG, leading to the degradation of HIF-1α, whereas overexpression of BCAT1 decreased α-KG concentrations, stabilizing HIF-1α and resulting in DNA hypermethylation through decreased TET activity. This hypermethylation is similar to that observed in AML cells with mutant isocitrate dehydrogenase (*IDH*), where TET2 is inhibited by 2-hydroxyglutarate, the product of mutant IDH. In AML the BCAT1-mediated changes in α-KG concentrations, and the consequent changes in HIF-1α stability and the epigenome, were thought to explain the association between increased BCAT1 expression and cell proliferation and disease progression. High concentrations of HIF-1α have also been implicated in the progression and maintenance of a tumor stem cell phenotype in glioblastoma.^53–55^ HIF-1α drives the expression of FOXM1,^39^ which is thought to be involved in maintaining a stem cell-like phenotype in glioblastoma cells.^40,43,45,46^ BCAT1 knockdown in A11 cells and xenografts decreased HIF-1α expression and in cells decreased in FOXM1 expression, which resulted in a significantly lower potential of the cells to form tumor spheroids.

BCAT1 overexpression, however, is evidently not universally essential for glioblastoma cell growth and invasion as indicated by the analysis of the TCGA dataset and the results obtained here with S2 cells. S2 cells had no measurable BCAT1 mRNA transcript or protein and yet had a doubling time that was similar to A11 cells and both had similar growth rates in vivo as xenografts.^16^ Single-cell RNA sequencing has also shown a subset of glioblastoma cells with very low BCAT1 expression.^23^ Unlike A11 cells, BCAT1 expression was not induced in S2 cells by hypoxia, although they have functional HIF-1α since overexpression of BCAT1 upregulated a transcriptional target of HIF-1α, CAIX, and they have a functional BCAT1 gene since ectopic expression of c-Myc drove expression of functional enzyme. The very low levels of BCAT1 in these cells can be explained by low levels of c-Myc. S2 cells are analogous to glioma cells expressing mutant *IDH*, where the production of 2HG is thought to silence BCAT1 expression.^7^

RNA-sequencing data indicates that glioblastoma cells exist in 4 main cellular states that recapitulate distinct neural cell types.^23^ More recently a pathway-based classification of glioblastoma also indicated the presence of 4 subtypes: Proliferative/progenitor, neuronal, mitochondrial, and glycolytic/plurimetabolic.^56^ Cells derived from mitochondrial subtype tumors, which are associated with a more favorable clinical outcome and increased sensitivity to radiotherapy, exhibited a higher basal oxygen consumption rate whereas cells derived from glycolytic subtype tumors, which are associated with a poor prognosis, exhibited a higher basal glycolytic rate. Tumors classified as mitochondrial were distributed across all 3 molecular subclasses identified previously (mesenchymal, proneural, and classical or proliferative), while glycolytic tumors were mostly mesenchymal.^57,58^ Epithelial-to-mesenchymal transition has been shown to drive BCAT1 expression,^22^ increased BCAT1 expression has been correlated with increased glycolytic enzyme expression^59^ and HIF-1α has been shown to drive migration, invasion, and mesenchymal marker expression in a glioblastoma cell line.^60^ RNA-sequencing data indicated that A11 belongs to a mesenchymal-like and S2 to a more neural progenitor cell-like state. Metabolic analyses in this and previous studies,^16^ and the demonstration that A11 cells have reduced sensitivity to radiotherapy than S2 cells,^16^ indicate that A11 is representative of the glycolytic mesenchymal subtype and S2 a mitochondrial subtype.

Overexpression of BCAT1 in both A11 and S2 cells increased HIF-1α expression but only promoted growth and invasion in A11 cells indicating that HIF-1α expression is not required for the growth of S2 cells, which appears to be driven primarily by c-Myc since overexpression of c-Myc in S2 cells (S2myc) promoted their proliferation. The inhibition of S2 cell proliferation following BCAT1 overexpression could be explained by their increased reliance on oxidative metabolism, where the decrease in α-KG concentration and increase in HIF-1α concentration resulting from BCAT1 overexpression will reduce TCA cycle flux,^61^ as was observed in A11 cells overexpressing BCAT1. The observation that the proliferation of S2 cells overexpressing c-Myc (S2myc), which increases BCAT1 expression, was inhibited by the BCAT1 inhibitor gabapentin suggests that when S2 cell proliferation is driven by increased c-Myc expression that this increased proliferation rate requires the increased BCAT1 activity.

In summary, increased expression of BCAT1 in glioblastoma cells with preexisting high levels of expression leads to a decrease in α-KG concentration, stabilization of HIF-1α, and increased cell proliferation and invasion mediated by HIF-1α-dependent expression of FOXM1. Conversely, increased BCAT1 expression in cells with constitutively low levels resulted in inhibition of proliferation despite increased HIF-1α expression. There are ongoing attempts to target BCAT1 for the treatment of glioblastoma.^62^ These observations suggest that stratification of tumors by BCAT1 expression may be necessary in order to select patients that could potentially respond to such therapeutic intervention.

## Supplementary Material

vdad120_suppl_Supplementary_Figures_S1-S6

## Data Availability

The data generated and code used in this study will be made publicly available in the University of Cambridge Apollo Repository.
