# Supplementary material for: The role of branched-chain aminotransferase 1 in driving glioblastoma cell proliferation and invasion varies with tumor subtype
Source: Neurooncol Adv. 2023 Sep 16;5(1):vdad120. doi: 10.1093/noajnl/vdad120 (PMC10599397; doi:10.1093/noajnl/vdad120)
Supplement: vdad120_suppl_Supplementary_Figures_S1-S6 [file vdad120_suppl_supplementary_figures_s1-s6.docx]

**SUPPLEMENTARY INFORMATION**

**The role of branched chain aminotransferase 1 in driving glioblastoma cell proliferation and invasion varies with tumor subtype**

Maria Fala^1^, Susana Ros^1^, Ashley Sawle^1^, Jyotsna U. Rao^1^, Anastasia Tsyben^1^, Laura Tronci^2^, Christian Frezza^2†^, Richard Mair^1^ and Kevin M. Brindle^1,3*^

^1^Cancer Research UK Cambridge Institute, University of Cambridge, Li Ka Shing Centre, Robinson Way, Cambridge, UK

^2^ MRC Cancer Unit, University of Cambridge, Hutchison/MRC Research Centre, Cambridge, UK,

^3^ Department of Biochemistry, University of Cambridge, Tennis Court Road, Cambridge, United Kingdom

current address

^†^Current address: CECAD Research Center, Faculty of Medicine, University Hospital Cologne Cologne, Germany

**Key resources table**

| REAGENT or RESOURCE | SOURCE | IDENTIFIER |
| --- | --- | --- |
| Antibodies | | |
| Rabbit polyclonal anti-BCAT1 | Cell Signaling Technology | Cat# 12822, RRID:AB_2798035 |
| Rabbit polyclonal anti-BCAT2 | Cell Signaling Technology | 9432, RRID:AB_2797701 |
| Mouse monoclonal anti-HIF1-alpha | Abcam | Cat# ab16066, RRID:AB_302234 |
| Rabbit polyclonal anti-HK-II | Cell Signaling Technology | Cat#2867; RRID: [AB_2232946](http://antibodyregistry.org/AB_2232946) |
| Rabbit monoclonal anti-FOXM1 | Cell Signaling Technology | Cat#5436: RRID: [AB_10692483](http://antibodyregistry.org/AB_10692483) |
| Rabbit polyclonal anti-c-Myc | Cell Signaling Technology | Cat# 9402, RRID:AB_2151827 |
| Rabbit polyclonal anti-Phospho-S6 (S235/236) | Cell Signaling Technology | Cat#2211; RRID: [AB_331679](http://antibodyregistry.org/AB_331679) |
| Rabbit polyclonal anti-CAIX | Novus Biologicals | Cat#NB100-417 |
| Mouse polyclonal anti-GAPDH | Sigma-Aldrich | Cat# G8795, RRID:AB_1078991 |
| Mouse polyclonal anti-beta actin | Sigma-Aldrich | Cat# A5441, RRID:AB_476744 |
| Rat monoclonal anti-tubulin | Abcam | Cat# ab6160, RRID:AB_305328 |
| Mouse monoclonal anti-BCAT1 | Proteintech | Cat# 67084-1-Ig, RRID:AB_2882392 |
| CAIX | BioScience Slovakia | Cat# AB1001 |
| MCT1 | Atlas | Cat# HPA003324, RRID:AB_1856982 |
| MCT4 | Atlas | Cat# HPA021451, RRID:AB_1853663 |
| Goat Horseradish Peroxidase (HRP) anti-Rabbit IgG | Cell Signaling Technology | Cat# 7074, RRID:AB_2099233 |
| Sheep Horseradish Peroxidase (HRP) anti-Mouse IgG | Cytiva | Cat# NA9310-1ml, RRID:AB_772193 |
| IRDye 800CW Goat anti-Rabbit IgG | Licor Biosciences | Cat# 925-32211, RRID:AB_2651127 |
| IRDye 680LT Goat anti-Mouse IgG | Licor Biosciences | Cat# 926-68050, RRID:AB_2783642 |
| Bacterial and Virus Strains | | |
| ONE SHOT STBL3 Competent Bacteria | ThermoFisher Scientific | Cat#C737303 |
| Chemicals, Peptides, and Recombinant Proteins | | |
| Doxycycline hyclate, dissolved in H2O | Sigma | Cat#D9891 |
| FAST Syber green Master Mix | ThermoFisher Scientific | Cat#4385610 |
| M-MuLV Reverse Transcriptase | NEB |  |
| Oligo(dT) 12-18 primer | ThermoFisher Scientific | Cat#18418012 |
| Protease inhibitor cocktail | Roche | Cat#11836170001 |
| Gibco DMEM | ThermoFisher Scientific | Cat# 21969035 |
| Gibco Neurobasal A | ThermoFisher Scientific | Cat# 12349015 |
| Gibco B27 | ThermoFisher Scientific | Cat#17504044 |
| N2 | ThermoFisher Scientific | Cat# A1370701 |
| Epidermal Growth Factor (EGF) | Sigma | Cat# E9644 |
| Fibroblast Growth Factor (FGF) | ThermoFisher Scientific | Cat # **PHG0261** |
| Gibco Penicillin - Streptomycin | ThermoFisher Scientific | Cat# 15070063 |
| Extracellular Matrix | Sigma | Cat# E1270 |
| Gibco StemPro Accutase Cell Dissociation Agent | ThermoFisher Scientific | Cat# A1110501 |
| Doxycyline diet (200 ppm) | Envigo | Cat# TD.180625 |
| Pierce RIPA buffer | Thermo Fisher Scientific | Cat#89901 |
| Leucine | Sigma-Aldrich | Cat# L602 |
| Α-ketoglutarate | Sigma-Aldrich | Cat# 75890 |
| Ammonium sulphate | Sigma-Aldrich | Cat# A4418 |
| Leucine dehydrogenase | Merck | Cat# 431525 |
| Gabapentin | Sigma-Aldrich | Cat# PHR1049 |
| DNA Degradase Plus | Zymo Research | Cat# E2020 |
| Critical Commercial Assays | | |
| Plasmid Maxi Kit | QIAGEN | Cat#12163 |
| RNAeasy kit | QIAGEN | Cat#74104 |
| Qubit RNA BR Assay kit | ThermoFisher Scientific | Q10210 |
| Shredders | QIAGEN | Cat#79654 |
| PCR Purification kit | QIAGEN | Cat#28104 |
| RealTime Glo assay kit | Promega | Cat#G9711 |
| **Experimental Models: Cell Lines** | | |
| U87 | ATCC | Cat# HTB-14, RRID:CVCL_0022 |
| U251 | ATCC | Cat# HTB-17, RRID:CVCL_2219 |
| A11 | Mair et al[1] | N/A |
| S2 | Mair et al [1] | N/A |
| SP20 | Mair et al [1] | N/A |
| A25 | This paper | N/A |
| **Experimental Models: Organisms/Strains** | | |
| BALB/c nude mice | Charles River | Strain Code: 194 (Homozygous) |
| RNU nude rats | Charles River | Strain Code: 316 (Homozygous) |
| Oligonucleotides | | |
| BCAT1 primers:  5’ CTGCCCCAGGTCTTGCTG 3’  5’ TGCAATCCTTCATTGTTCCGTC 3’ | This paper | N/A |
| BCAT2 primers:  5’ GGGCAGATCTGGGCACG 3’  5’ GTCTGCAGCCTTGAAACTGG 3’ | This paper | N/A |
| BCAT1_KD: shRNA: 5' - CCGGCCCAATGTGAAGCAGTAGATACTCGAGTATCTACTGCTTCACATTGGGTTTTT - 3’ | Tönjes et al[2] | N/A |
| BCAT2_KD: shRNA: 5’ - CCGGACTACAAGTTAGGTGGGAATTCTCGAGAATTCCCACCTAACTTGTAGTTTTTTG - 3’ | This paper | N/A |
| Control shRNA: 5′-CCGGCCTAAGGTTAAGTCGCCCTCGCTCGAGCGAGGGCGACTTAACCTTAGG | Mair et al [1] | N/A |
| **Recombinant DNA** | | |
| TetOnPLKO.puro | Wiederschain et al [3] | Addgene: #21915; RRID: Addgene_21915 |
| pBOBI | Verma laboratory, Salk Institute La Jolla, USA | N/A, gift to K.M.B |
| **Software and Algorithms** | | |
| FIJI 2.0.0-rc-69/1.52p Image J | ImageJ | <https://imagej.net/Fiji>; RRID: SCR_002285 ImageJ |
| R | R Core Team | <https://www.r-project.org/>; RRID: SCR_001905 |
| GraphPad Prism 9.0 | GraphPad Prism | RRID:SCR_002798 |

**Supplementary Methods**

**MRI**

Proton images were acquired using a 7.0 T scanner (Agilent, Palo Alto, US). Forty-two and 72 mm diameter coils were used for mice and rats respectively. Animals were anaesthetized by inhalation of 2-3% isoflurane in air/O_2_ (75/25% vol/vol, 2 L/min). Respiration and body temperature were monitored over the course of the experiment and temperature maintained using a stream of warm air. T_2_-weighted images were acquired from 8 echoes using a Fast Spin Echo sequence, with 15 axial slices, 40 mm x 40 mm field-of-view, 2 mm slice thickness, a matrix size of 128 x 128, a TR of 1.8 s and a TE of 50 ms.

**Spheroid Invasion Assay Data Analysis**

Data were analyzed using a Python script that used contrast enhancement (Contrast Limited Adaptive Histogram Equalization), denoising (Non-local means denoising) and thresholding tools. The areas containing cells were identified by measuring the standard deviation within small contiguous regions.

**RNA sequencing data analysis**

Single end 50 nt reads were aligned to the Ensembl GRCh38 genome using STAR version 2.5.3a [4] with default parameters. Counts of reads against genes were generated using featureCounts from the Subread package version 1.5.2 [5] and only primary alignments were counted. Differential gene expression analysis was carried out in R version 3.5.2 [6] using the DESeq2 package version 1.5.2 [7] with default parameters. Multiple testing correction of p-values was carried out using the Benjamini-Hochberg method [8]. Genes were determined to be statistically differentially expressed at an adjusted p-value of 0.05.

**Enrichment of HIF1α and FOXM1 targets**

Gene Set Enrichment Analysis [9] was performed using the package clusterProfiler (v 4.2.2) [10] to assess enrichment of HIF1α and FOXM1 target genes in the differentially expressed genes. Lists of HIF1α and FOXM1 targets from Lachmann et al (2010) [11] were downloaded from the authors’ website (<https://maayanlab.cloud/Harmonizome/>). The Wald test statistic from the DESeq2 analysis was used to pre-rank the genes.

**Expression of BCAT1 in IDH wild-type Glioblastoma in TCGA**

RNA-seq gene expression quantification files for 174 TCGA glioblastoma samples were downloaded using the GDC Data Transfer Tool. Of these 138 were identified as IDH wild-type primaries using the R packages TCGAutils version 1.18.0 [12] and curatedTCGAData version 1.20.0 [13] . Raw count data for these samples was combined with raw count data for 8 A11 PDX samples and 4 S2 PDX samples. Data were normalised across samples using the *vst* (variance stabilising transformation) function from the DESeq2 package. BCAT1 expression for each data set was visualised using the R package ggplot2 [14].

**Tumor classification based on RNA sequencing**

Raw reads from tumor RNA sequencing were separated into rat (host) and human (tumor) reads using Xenome [15], the GRCh38 human genome assembly from Ensembl and the Rnor_6.0 *Rattus norvegicus* genome assembly from Ensembl. Gene expression in the patient-derived tumors was quantified using Salmon [16] and a reference derived from the Ensembl GRCh38 release 102 transcriptome. Neftel et al [17] described four cellular states OPC-like, NPC-like, AC-like and MES-like in glioblastoma samples and described a method for scoring based on gene expression. For each PDX sample, read counts from Salmon were TPM normalised and cellular state scores were calculated as described in Neftel et al [17] using code provided by the corresponding author.

**LC-MS of intracellular metabolites**

HILIC chromatographic separation of metabolites was achieved using a Millipore Sequant ZIC-pHILIC analytical column (5 µm, 2.1 × 150 mm) equipped with a 2.1 × 20 mm guard column with a binary solvent system. Solvent A was 20 mM ammonium carbonate, 0.05% ammonium hydroxide; Solvent B was acetonitrile. The column oven and autosampler tray were held at 40 °C and 4 °C, respectively. The chromatographic gradient was run at a flow rate of 0.2 mL/min as follows: 0–2 min: 80% B; 2-17 min: linear gradient from 80% B to 20% B; 17-17.1 min: linear gradient from 20% B to 80% B; 17.1-22.5 min: hold at 80% B. Samples were randomized and analysed with LC–MS in a blinded manner with an injection volume of 5 µl. Metabolites were measured with a Thermo Scientific Q Exactive Hybrid Quadrupole-Orbitrap Mass spectrometer (HRMS) coupled to a Dionex Ultimate 3000 UHPLC. The mass spectrometer was operated in full-scan, polarity-switching mode, with the spray voltage set to +4.5 kV/-3.5 kV, the heated capillary held at 320 °C, and the auxiliary gas heater held at 280 °C. The sheath gas flow was set to 25 units, the auxiliary gas flow was set to 15 units, and the sweep gas flow was set to 0 unit. HRMS data acquisition was performed in a range of m/z = 70–900, with the resolution set at 70,000, the AGC target at 1 × 10^6^, and the maximum injection time (Max IT) at 120 ms. Metabolite identities were confirmed using two parameters: (1) precursor ion m/z was matched within 5 ppm of theoretical mass predicted by the chemical formula; (2) the retention time of a metabolite was within 5% of the retention time of a purified standard run with the same chromatographic method. Chromatogram review and peak area integration were performed using the Thermo Fisher software Tracefinder 5.0 and the peak area for each detected metabolite was normalized against the total ion count (TIC) of that sample to correct for any variations introduced from sample handling through to instrument analysis. The normalized areas were used as variables for further statistical data analysis. Absolute quantification of αKG concentration was performed by interpolation of the corresponding standard curve obtained from serial dilutions of the commercially available standard (Sigma Aldrich) run with the same batch of samples using the method described above.

**Measurement of 5-hydroxymethylcytosine**

The LC-MS system consisted of a Shimadzu Nexera X2 UHPLC and Sciex 6500 Triple Quad mass spectrometer, running reversed phase chromatography using a Phenomenex Kinetex 1.7µm Biphenyl 100Å 100x2.1 mm column at 30°C. Samples were stored at 6°C within the LC autosampler. A gradient of 0.1% formic acid in water and an increasing concentration of 0.1% formic acid in acetonitrile over 3 minutes at 300 µL/min was used to retain and separate the analytes of interest, with a further 5 minutes of column re-equilibration. The mass spectrometer was run in positive electrospray ionization mode and each analyte’s MRM transition at unit resolution was monitored. Samples were injected alongside calibration standards and batch acceptance QCs. Chromatograms were processed and integrated using Sciex MultiQuant software.

**LC-MS for labeled leucine enrichment**

Plasma samples were thawed on wet ice and an aliquot was diluted 50-fold using cold methanol:acetonitrile:water (50:30:20), mixed and centrifuged at 21,100 g for 10 min at 4°C, and the supernatant transferred to a fresh tube and frozen at -80°C for a minimum of overnight storage. Before analysis, the supernatant was centrifuged again at 21,100 g for 10 min at 4°C and an aliquot transferred to a 96 well plate. Frozen tissue samples were weighed and homogenized in cold methanol:acetonitrile:water (50:30:20) at a concentration of 100 mg/mL using a Precellys Cryolys Evolution homogenizer at 4°C. The homogenate was then centrifuged at 21,100 g for 10 min at 4°C, the supernatant was transferred to a fresh tube and frozen at -80°C for a minimum of overnight storage. Before analysis the supernatant was centrifuged again at 21,100 g for 10 min at 4°C and then an aliquot was further diluted to 20 mg/mL (so that the metabolite concentrations were approximately equivalent to those in the plasma samples) and an aliquot transferred to a 96 well plate. An aliquot of each sample was pooled to make a biological quality control for repeated injection throughout the batch acquisition, in order to monitor the system’s consistency. A standard solution containing the relevant metabolites was prepared for injection before and after the batch acquisition to confirm detection, retention time and peak shape of the metabolites. The LC-MS system consisted of a Shimadzu Nexera X2 UHPLC and Sciex 6600 Triple TOF mass spectrometer, running HILIC chromatography using a SeQuant ZIC-pHILIC 5µm 150 x 2.1mm column (with Sequant ZIC-pHILIC 20 x 2.1mm guard column) at 45°C. Samples were stored at 6°C within the LC autosampler. Each sample was injected twice for analysis in both positive and negative electrospray ionization TOFMS to cover the metabolites required within the scan range of 75-1000 Da. The two mobile phases used were 20 mM ammonium carbonate pH 9.4 and 100% Acetonitrile. A gradient of these phases was used over 17 minutes at 200 µL/min to retain and separate the metabolites of interest, with a further 11.5 minutes of column re-equilibration. The mass spectrometer was calibrated after every 4^th^ injection using appropriate polarity Sciex calibration solution.

**Supplementary figures**

**
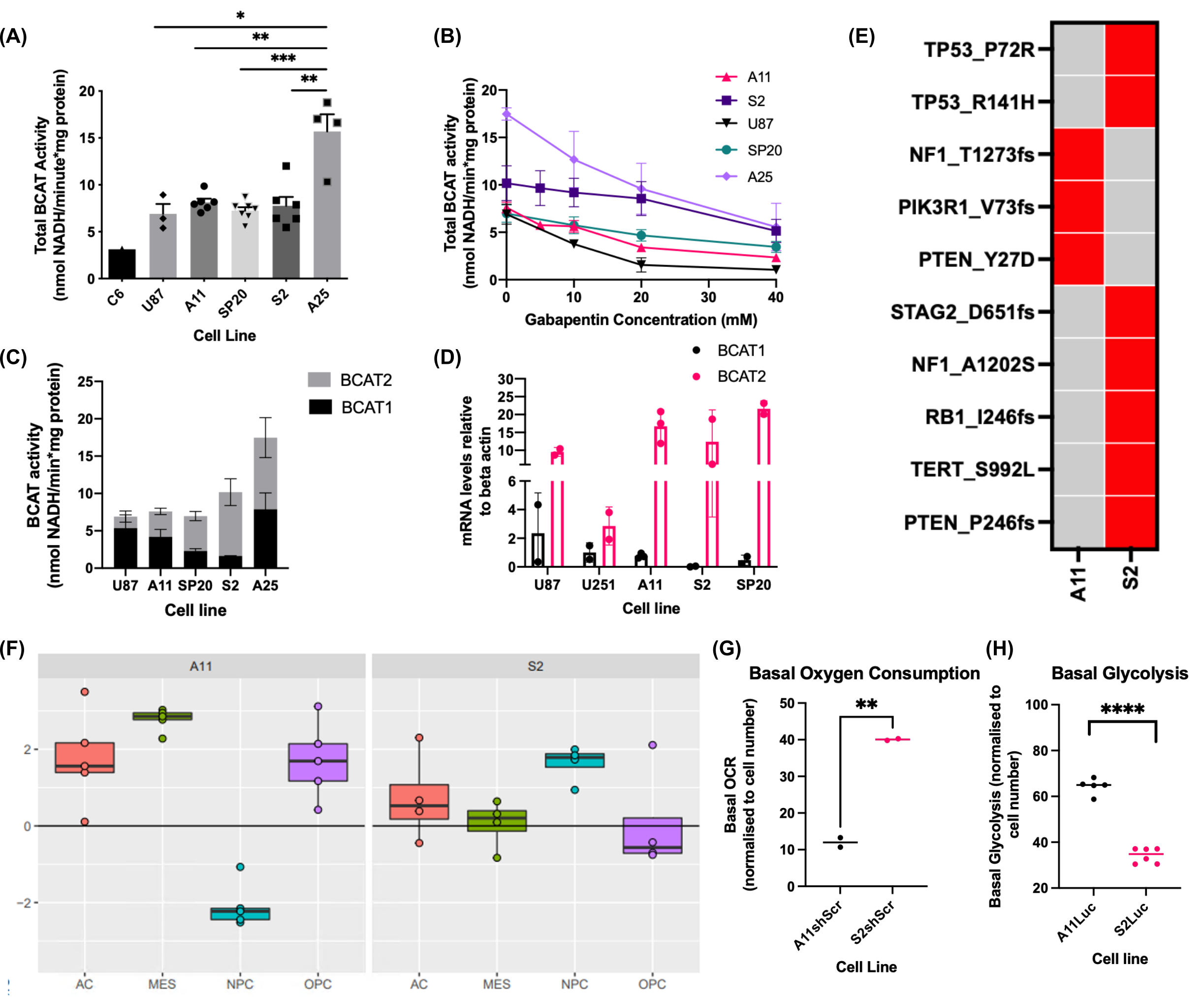
**

**Figure S1:** (A) Spectrophotometric measurements of total BCAT activity in cell extracts. Each point represents a biological replicate and error bars represent Standard Errors of the Mean (SEM). Two-tailed t-tests were used to compare the total BCAT activity between different cell lines. *p<0.05, **p<0.01, ***p<0.001. (B) Increasing concentrations of gabapentin, a selective inhibitor of BCAT1, were added to the spectrophotometric assay to discriminate between BCAT1 and BCAT2 activities. Error bars represent SEM. (C) Estimated contributions of BCAT1 and BCAT2 to the total activity as measured in a spectrophotometric assay, calculated with the assumption that 20 mM gabapentin inhibits all of the BCAT1 activity without affecting BCAT2 activity. Error bars represent SEM. (D) RT-qPCR measurements of BCAT1 and BCAT2 mRNA expression relative to β-actin. Each point represents a biological replicate and error bars represent Standard Deviations. (E) Mutations identified from Whole Exome Sequencing of A11 and S2 cells. Red squares represent presence of the indicated mutation and grey squares represent absence of mutation. Abbreviation: fs- frameshift mutation. (F) RNA sequencing based classification of A11 and S2 rat orthotopic xenograft samples based on Neftel et al classification [17]. Each point represents a biological replicate. Abbreviations: AC- Astrocyte-like, MES – Mesenchymal, NPC – Neural Progenitor Cell, OPC – Oligodendocytic Precursor Cell. (G-H) Seahorse metabolic assays comparing A11 and S2 cells’ basal oxygen consumption rate (G) and basal glycolysis rate (H). Two-tailed t-tests were performed to compare the two cell lines **p<0.01, ****p<0.0001.


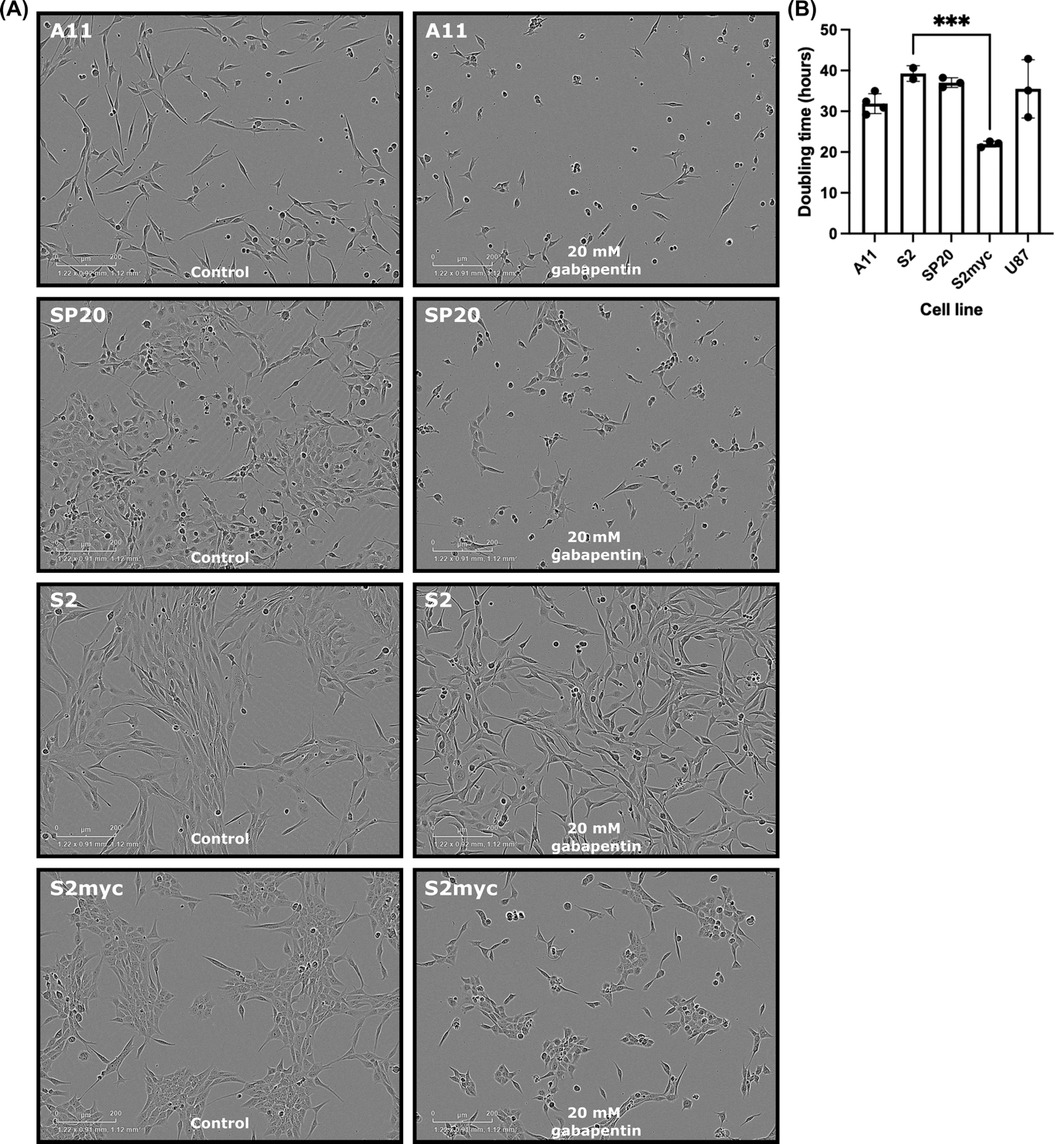


Figure S2: (A) Representative images showing changes in morphology of A11, SP20, S2 and S2myc cells following the addition of 20 mM gabapentin. (B) Doubling times of A11, S2, SP20, S2myc and U87 cells, as calculated from changes in cell confluence measured using an Incucyte scanner. Each point represents a biological replicate and the error bars represent Standard Deviations. A two-tailed t-test was used to compare S2 and S2myc cells (***p<0.001).


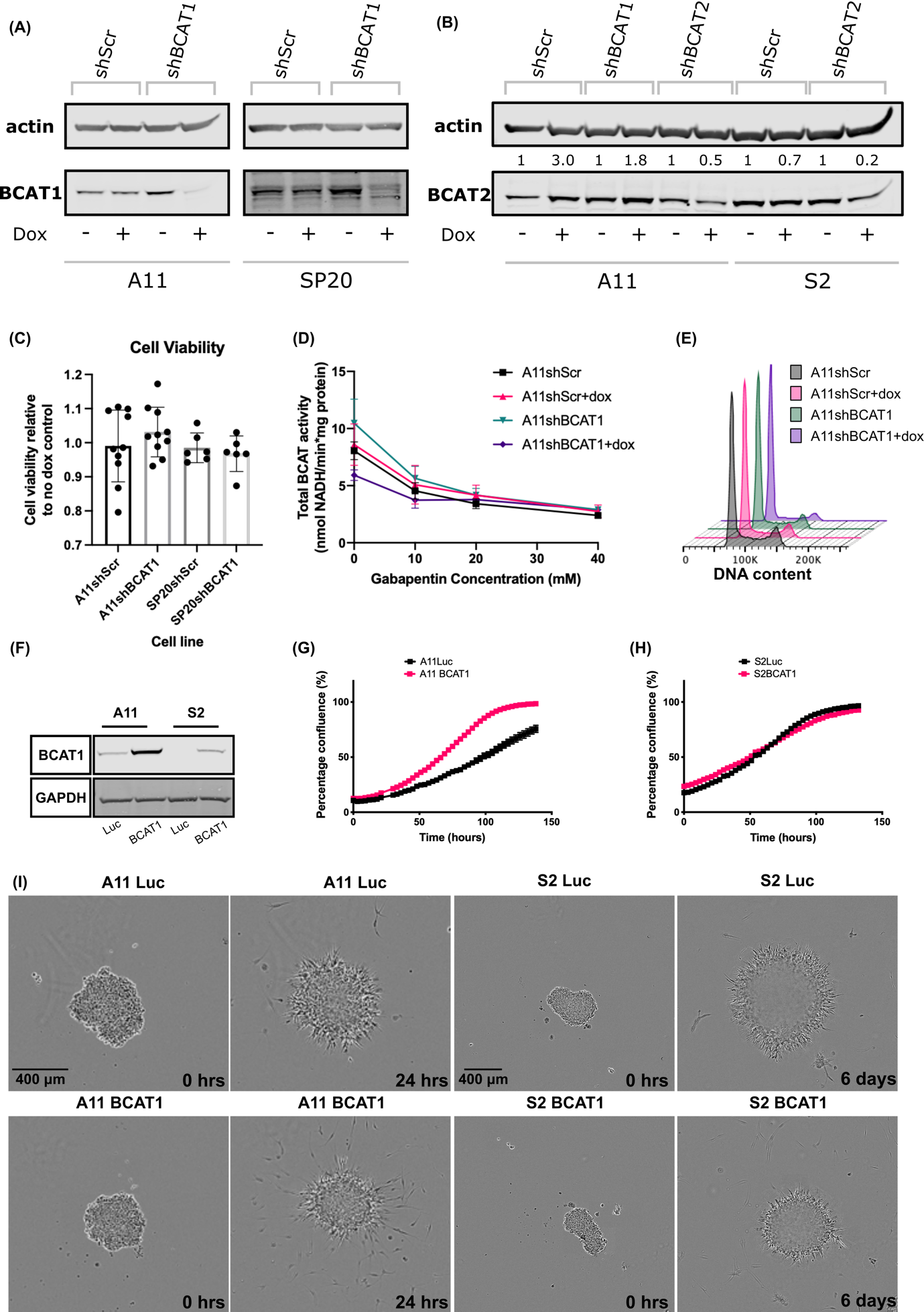


**Figure S3:** (A) Western blot for BCAT1 in A11 and SP20 cells expressing doxycycline-inducible shScr and shBCAT1. Dox: doxycycline. β-actin was used as a loading control. (B) Western Blot for BCAT2 in A11 and S2 cells expressing doxycycline-inducible shScr and shBCAT2. The numbers above the BCAT2 bands correspond to BCAT2 expression relative to the uninduced control. (C) Cell viability in A11 and SP20 cells expressing doxycycline-inducible shScr and A11shBCAT1 relative to their respective no doxycycline controls, as measured using Trypan Blue dye exclusion. (D) Total BCAT activity measured in lysates of A11shScr and A11shBCAT1 cells treated with doxycycline and their respective no doxycycline treatment controls in the presence of increasing concentrations of gabapentin in the enzyme assay. Error bars represent Standard Error of the Mean (n=2). Two-tailed t-tests were used to compare the BCAT1 activities in the different cell lines.

(E) Representative example of the distribution of cells in the cell cycle phases, with histograms normalized to the mode. (F) Representative western blot for BCAT1 in luciferase-overexpressing and BCAT1-overexpressing A11 and S2 cells. GAPDH was used as a loading control. Luc: Luciferase. (G-H) Representative proliferation curves for A11 (G) and S2 (H) cells overexpressing luciferase and BCAT1

(I) Representative images of A11 and S2 luciferase-overexpressing neurospheres and BCAT1-overexpressing neurospheres at the time of embedding in matrigel (0 hrs) and 24 hours or six days later.

**
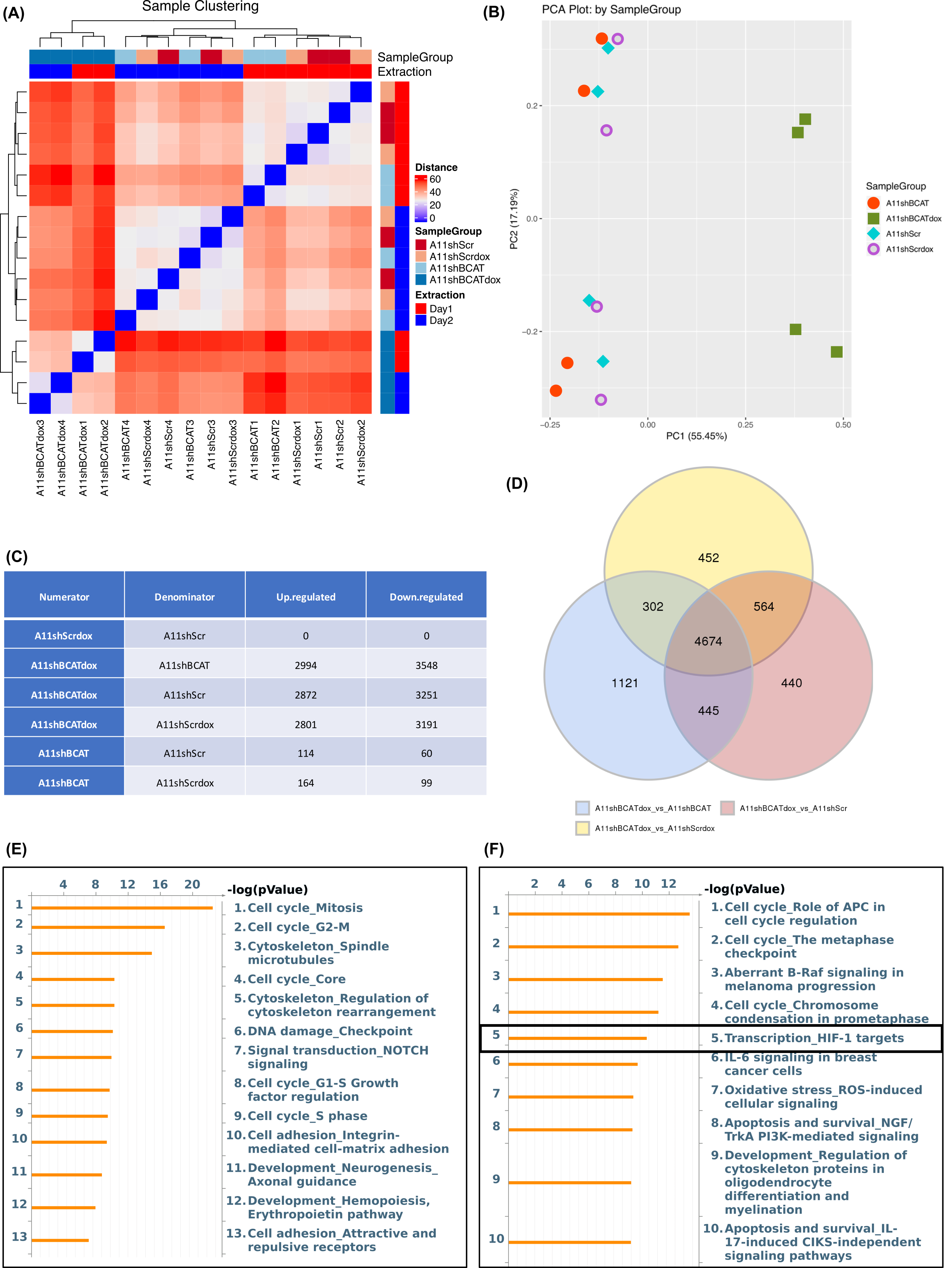
**

**Figure S4: BCAT1 knockdown leads to changes in the transcriptional profile of A11 cells.** (A) Heatmap illustrating clustering analysis of RNA sequencing samples, based on the pairwise distance between samples, considering read counts at all annotated features. (B) Principal Component Analysis showing the first two principal components of the variance-stabilizing transformed raw counts, summarizing the relationship among the samples, based on the top 500 most variable features (genes with counts that show highest variance across samples). (C) Table summarizing the number of differentially expressed genes in comparisons between the different samples. (D) The overlap of differentially expressed genes when comparing doxycycline-induced A11 cells expressing shBCAT1 to each of three control groups (A11shScr, doxycycline-induced A11shScr and A11shBCAT1). (E-F) Gene enrichment analysis results using the Metacore tool (Clarivate Analytics). The list includes genes that were differentially expressed in both comparisons of A11shBCAT1+dox transcripts with A11shBCAT1 without dox and A11shScr+dox transcripts. The top process networks (E) and pathway maps (F) that were enriched in the gene list are plotted here against their respective -log(pValue). Transcription of HIF target genes was identified as an enriched pathway map (outlined by the black box).

**
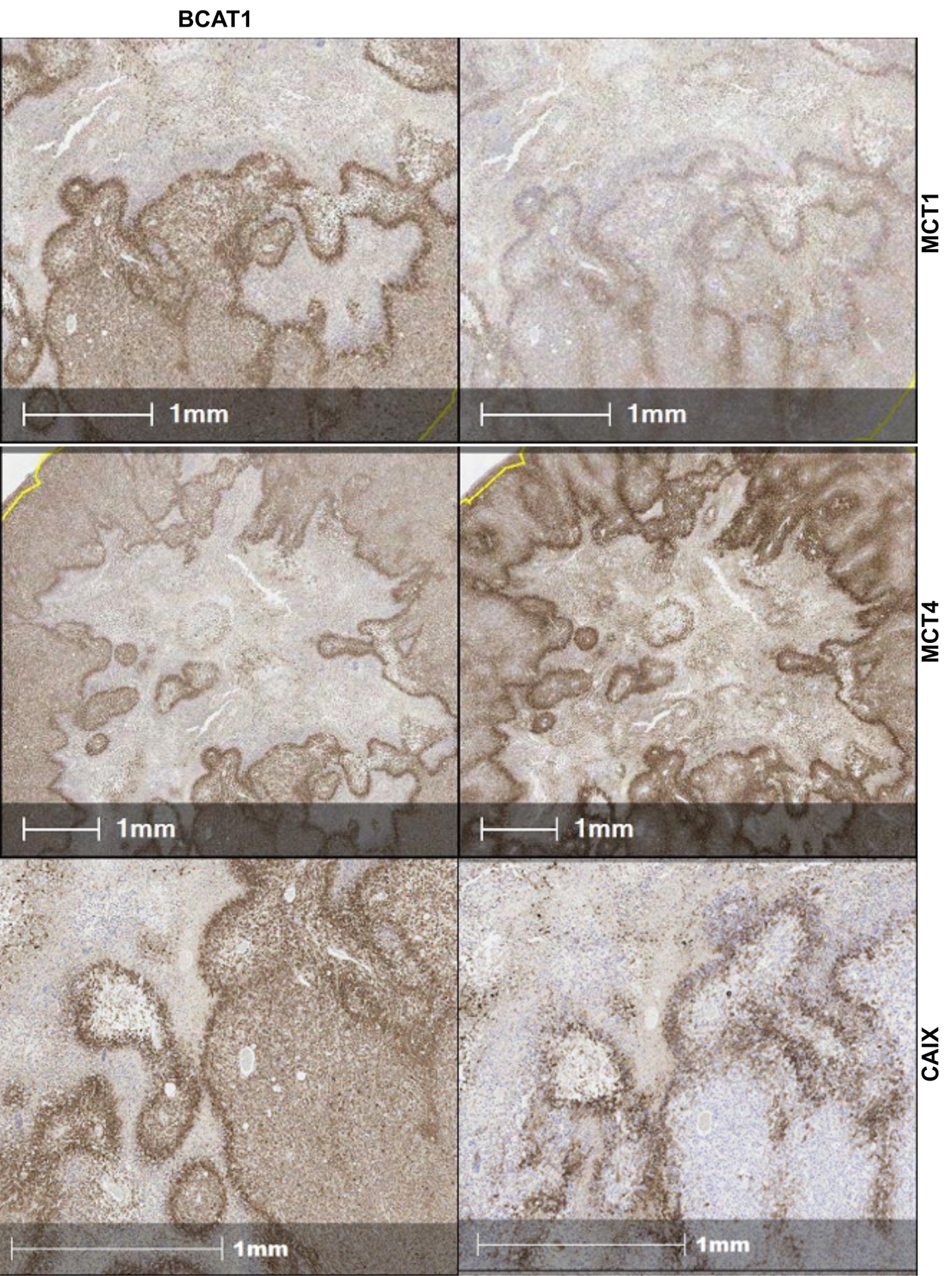
**

**Figure S5: BCAT1 regulates the expression of HIF targets.** Representative immunohistochemical images of a xenograft in a rat produced by orthotopic implantation of A11 cells. Images on the left show BCAT1 staining and co-registered images on the right show staining for MCT1, MCT4 and CAIX.


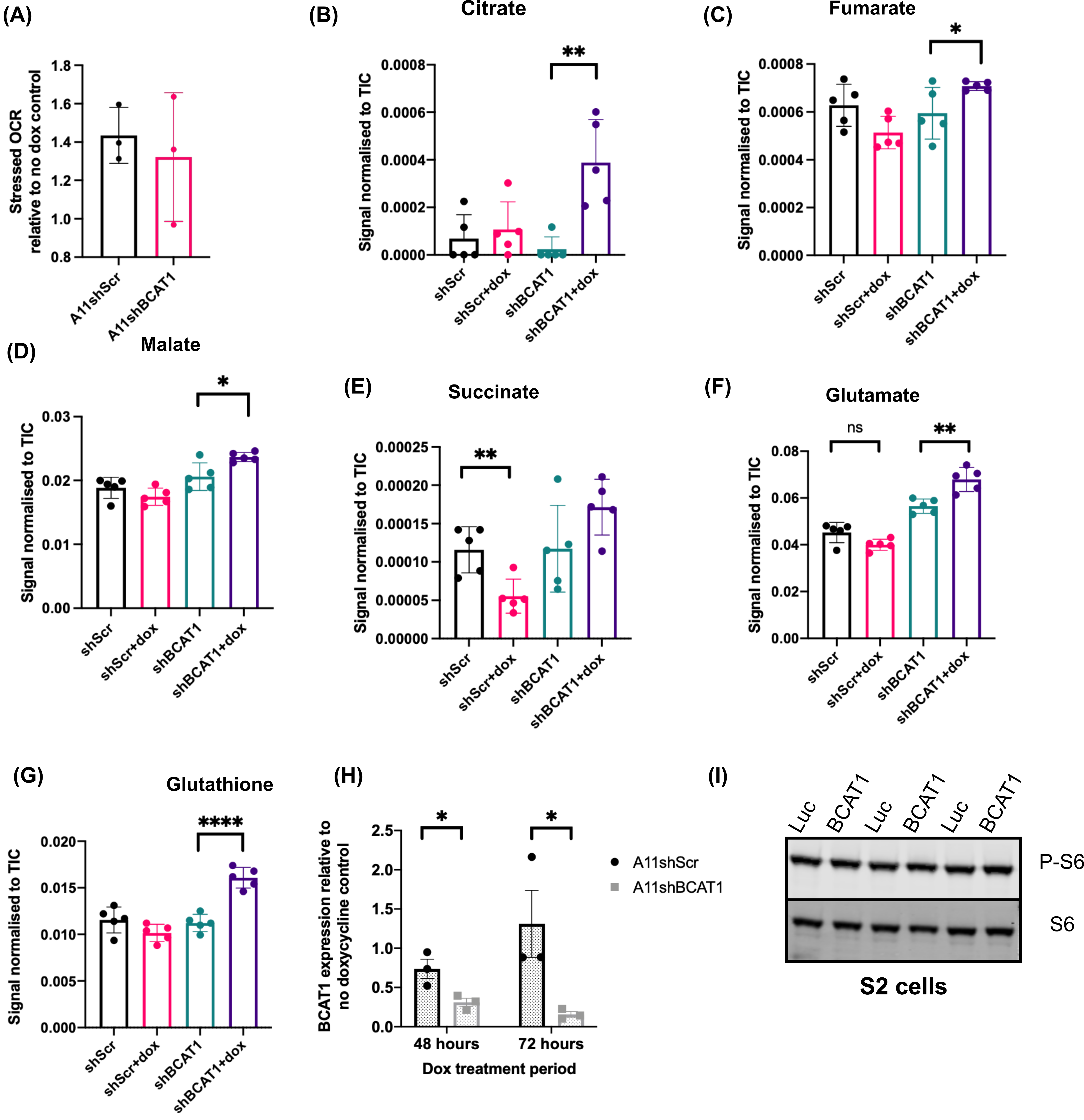


**Figure S6:**

(A) Stressed Oxygen Consumption Rates (OCR) following the addition of FCCP to A11 cells expressing doxycycline-inducible shScr and shBCAT1 relative to their respective no doxycycline treatment controls. Each point represents the mean from an independent experiment. Error bars represent Standard Deviations. (B-G) The relative citrate (B), fumarate (C), malate (D), succinate (E), glutamate (F) and glutathione (G) concentrations in extracts of A11 cells expressing doxycycline-inducible shScr and shBCAT1 and their respective no doxycycline treatment controls. Error bars represent Standard Deviations. Two-tailed t-tests were used to compare the different groups, ns: p>0.05, *p<0.05, **p<0.01, ****P<0.0001. (H) BCAT1 protein content in A11 cells expressing doxycycline-inducible shScr and shBCAT1, relative to their respective no doxycycline treatment controls, following 48 and 72 hours of doxycycline treatment. Two-tailed t-tests were used to compare shScr- and shBCAT1-expressing cells. *p<0.05. (I) Western Blots of phosphorylated S6 (PS6) and total S6 in lysates from BCAT1 and luciferase overexpressing S2 cells.

**References:**

1. Mair, R., et al., *Metabolic imaging detects low levels of glycolytic activity that vary with levels of c-Myc expression in patient-derived xenograft models of glioblastoma.* Cancer research, 2018. **78**(18): p. 5408-5418.

2. Tönjes, M., et al., *BCAT1 promotes cell proliferation through amino acid catabolism in gliomas carrying wild-type IDH1.* Nature medicine, 2013. **19**(7): p. 901-908.

3. Wiederschain, D., et al., *Single-vector inducible lentiviral RNAi system for oncology target validation.* Cell Cycle, 2009. **8**(3): p. 498-504.

4. Dobin, A., et al., *STAR: ultrafast universal RNA-seq aligner.* Bioinformatics, 2013. **29**(1): p. 15-21.

5. Liao, Y., G.K. Smyth, and W. Shi, *featureCounts: an efficient general purpose program for assigning sequence reads to genomic features.* Bioinformatics, 2014. **30**(7): p. 923-30.

6. Team, R.C., *R: A language and environment for statistical computing*, in *R Foundation for Statistical Computing*. 2018.

7. Love, M.I., W. Huber, and S. Anders, *Moderated estimation of fold change and dispersion for RNA-seq data with DESeq2.* Genome Biology, 2014. **15**(12): p. 550.

8. Benjamini, Y. and Y. Hochberg, *Controlling the False Discovery Rate: A Practical and Powerful Approach to Multiple Testing.* Journal of the Royal Statistical Society: Series B (Methodological), 1995. **57**(1): p. 289-300.

9. Subramanian, A., et al., *Gene set enrichment analysis: a knowledge-based approach for interpreting genome-wide expression profiles.* PNAS, 2005. **102**(43): p. 15545-15550.

10. Wu, T., et al., *clusterProfiler 4.0: A universal enrichment tool for interpreting omics data.* The Innovation, 2021. **2**(3): p. 100141.

11. Lachmann, A., et al., *ChEA: transcription factor regulation inferred from integrating genome-wide ChIP-X experiments.* Bioinformatics, 2010. **26**(19): p. 2438-2444.

12. Ramos, M., L. Schiffer, and L. Waldron, *TCGAutils: TCGA utility functions for data management. R package version 1.18.0*. 2022.

13. Ramos, M., et al., *Multiomic integration of public oncology databases in bioconductor.* JCO Clinical Cancer Informatics, 2020. **1**: p. 958-971.

14. Wickham, H., *Data analysis*, in *ggplot2*. 2016, Springer. p. 189-201.

15. Conway, T., et al., *Xenome--a tool for classifying reads from xenograft samples.* Bioinformatics, 2012. **28**(12): p. i172-8.

16. Patro, R., et al., *Salmon provides fast and bias-aware quantification of transcript expression.* Nat Methods, 2017. **14**(4): p. 417-419.

17. Neftel, C., et al., *An Integrative Model of Cellular States, Plasticity, and Genetics for Glioblastoma.* Cell, 2019. **178**(4): p. 835-849.e21.
